# Optimal myelin elongation relies on YAP activation by axonal growth and inhibition by Crb3/Hippo pathway

**DOI:** 10.1038/ncomms12186

**Published:** 2016-07-20

**Authors:** Ruani N. Fernando, Laurent Cotter, Claire Perrin-Tricaud, Jade Berthelot, Sylvain Bartolami, Jorge A. Pereira, Sergio Gonzalez, Ueli Suter, Nicolas Tricaud

**Affiliations:** 1 Institut des Neurosciences de Montpellier, INSERM U1051, University of Montpellier, Hopital Saint Eloi, 80 rue A. Fliche, 34090 Montpellier, France; 2 ETH, Institute of Molecular Health Sciences, HPL F 36 Otto-Stern-Weg 7, 8093 Zürich, Switzerland

## Abstract

Fast nerve conduction relies on successive myelin segments that electrically isolate axons. Segment geometry—diameter and length—is critical for the optimization of nerve conduction and the molecular mechanisms allowing this optimized geometry are partially known. We show here that peripheral myelin elongation is dynamically regulated by stimulation of YAP (Yes-associated protein) transcription cofactor activity during axonal elongation and limited by inhibition of YAP activity via the Hippo pathway. YAP promotes myelin and non-myelin genes transcription while the polarity protein Crb3, localized at the tips of the myelin sheath, activates the Hippo pathway to temper YAP activity, therefore allowing for optimal myelin growth. Dystrophic *Dy*^*2j/2j*^ mice mimicking human peripheral neuropathy with reduced internodal lengths have decreased nuclear YAP which, when corrected, leads to longer internodes. These data show a novel mechanism controlling myelin growth and nerve conduction, and provide a molecular ground for disease with short myelin segments.

## Abstract (web summary)

Molecular mechanisms regulating optimal myelin geometry are only partially understood. Here authors show that peripheral myelin growth is orchestrated by the Crb3/Hippo/YAP pathway, and that defects in YAP activation may underlie peripheral neuropathies caused by shorter myelin.

## Graphical abstract

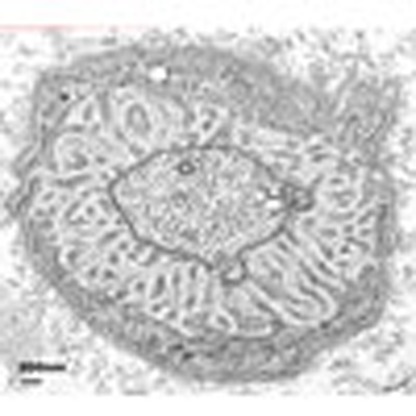

Fast nerve conduction relies on successive myelin segments that electrically isolate axons between nodes of Ranvier. The myelin geometry—diameter and internodal length—is therefore a critical parameter of the nerve conduction velocity and changes in this geometry underlie numerous functional properties of the brain and nerves^1^,^2^. Moreover reduced internodal length, which strongly impairs nerve conduction velocity^3^,^4^,^5^, is linked to human neuropathies such as multiple sclerosis in the central nervous system^5^ and CMT4F (*Periaxin* gene mutations)^6^,^7^,^8^ and congenital neuro-muscular dystrophy 1A (*α2 laminin* gene mutations)^9^ in the peripheral nervous system. While the molecular mechanisms responsible for the homogeneity of myelin thickness in Schwann cells along the same axon have been uncovered^10^,^11^,^12^, the mechanisms responsible for myelin elongation and the formation of regular myelin lengths remain unknown. Using the remarkable homogeneity of myelin internodes along axons of the peripheral nervous system we investigated these mechanisms *in vivo* and *in vitro*.

Recently the Hippo pathway and YAP (Yes-associated protein) transcription cofactor have been shown to be perturbed in Schwannomas, suggesting that they play an unknown role in Schwann cells^13^. Here we show that myelin elongation in Schwann cells is dynamically regulated by the stimulation of YAP activity during the nerve elongation that occurs with postnatal body growth. This elongation is finely tuned as YAP activity is simultaneously inhibited by the activation of the Hippo pathway, initiated by the transmembrane polarity protein Crb3 at the node of Ranvier. The relevance of YAP was confirmed in dystrophic *Dy*^*2j/2j*^ mice that mimic a human peripheral neuropathy with reduced internodal lengths^9^. We found that the shortened myelinating Schwann cells (mSCs) have depleted nuclear YAP which when corrected, by lifting the Crb3/Hippo inhibition, leads to longer internodes. We conclude that the Crb3/Hippo/YAP pathway is a major regulator of myelination in peripheral nerves and we propose that defects in YAP activation underlie peripheral neuropathies with reduced internodal length. The modulation of this pathway can be used to correct myelination and myelin geometry.

## Results

### Crb3 negatively regulates myelin sheath elongation

**Figure 1:**
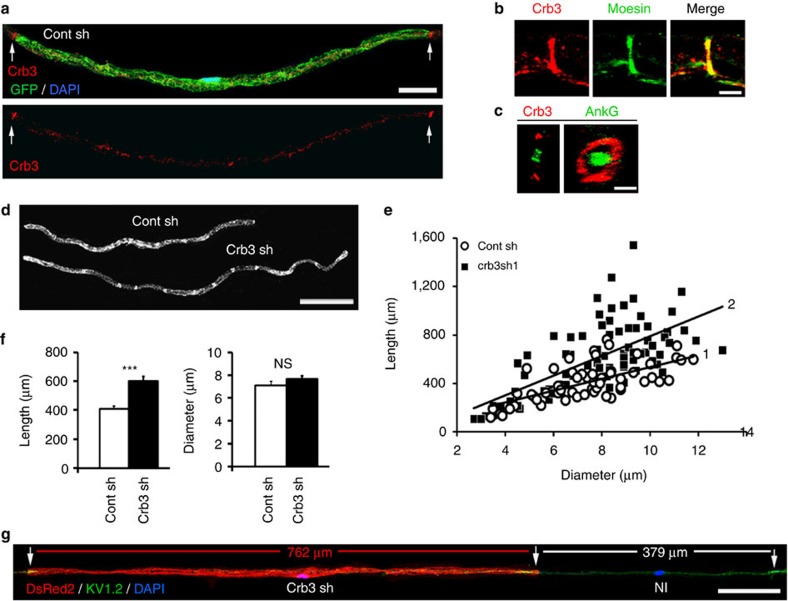
Crb3 negatively regulates myelin sheath elongation. (**a**) Crb3 immunostaining (red) localizes as discrete lines typical of microvilli at the extremities of mSC (GFP, green). (**b**) Crb3 (red) partially colocalizes with microvilli marker moesin (green) in a typical band pattern. (**c**) Confocal images *Z* axis projection of Crb3 immunostaining (red) reveals a typical microvilli ring around axonal node marker Ankyrin G (green). (**d**) Representative mSCs infected with Crb3 (Crb3 sh, DsRed2) or control shRNAs (Cont sh, GFP) lentiviruses. (**e**) Lengths versus diameters of mSCs infected with Crb3 (black square, *n*=83, slope line 2) or control (white circle, *n*=60, slope line 1) shRNA viruses. Two independent shRNAs against Crb3 yielded similar results (results from only one is shown). Three animals for control and Crb3 sh virus. (**f**) Average length (left) and diameter (right) of mSCs described in **e**. (**g**) mSC infected with Crb3 shRNA virus (Crb3sh, DsRed2) is longer than the next non-infected mSC (NI) on the same axon. Nodal regions (arrows) are visualized using Kv1.2 immunostaining (green) and nuclei using DAPI (blue). Statistical two-tailed Student's *t*-tests: ***P<0.001; NS, *P*>0.05 not significant. Scale bars, 100 μm (**a**,**d**,**g**), 5 μm (**b**,**c**). Error bars show s.e.m.

We found that the apical polarity protein Crb3, one of the mammalian homologues of drosophila Crumbs^14^, is expressed in the peripheral nerve (Supplementary Fig. 1) and specifically distributes to the microvilli of mSCs. These microvilli are fine cellular protrusions that originate from the outer membrane of the Schwann cell, mix with microvilli of neighbouring cells and contact the node of Ranvier (Supplementary Fig. 2a,e). Crb3 immunostaining on teased fibres of mouse sciatic nerves appeared as two lines at the extremities of the mSC (Fig. 1a) where it colocalized with known microvilli marker moesin (Fig. 1b). In a single confocal plane Crb3 staining localized either side of the axonal membrane (Supplementary Fig. 2b) and when confocal scans were projected on the *z* axis Crb3 staining appeared as a ring surrounding the node of Ranvier (Fig. 1c), characteristic of microvilli structure. A minor Crb3 staining was also observed along the internode at the inner edge, adaxonal domain of the myelin sheath (Supplementary Fig. 2c–e).

To address the role of Crb3 in mSC microvilli we designed lentiviral vectors expressing mouse Crb3 shRNAs (Supplementary Fig. 3a) or control shRNA under the control of a U6 promoter together with DsRed2 under a CMV promoter. We injected these viruses into sciatic nerves of mouse pups at postnatal days (dPN) 3–5, when myelination starts^10^,^15^. Under these conditions lentiviral vectors infect mSCs but not neurons^16^. Two months later—when myelination is near completion—these mice were killed and the injected sciatic nerves were fixed. Myelinated fibres were teased on glass slides, immunostained and analysed using confocal microscopy. As expected infected mSCs expressing DsRed2 and Crb3 shRNA expressed less Crb3 than non-infected cells (Supplementary Fig. 3b,c). We also observed that mSCs silenced for Crb3 were much longer than control cells (Fig. 1d–f), suggesting that Crb3 plays an inhibitory role during myelination. Indeed on the same axon Crb3-silenced cells were typically longer than non-infected neighbouring cells (Fig. 1g). However the fibre diameter did not change (Fig. 1f). We also attempted to overexpress Crb3 in mSCs *in vivo*, however this induced a strong demyelinating phenotype that prevented the analysis of the myelin length (Supplementary Fig. 3d).

To check whether Crb3 silencing affected the myelin sheath ultrastructure we replaced DsRed2 with placental alkaline phosphatase (PLAP) in our lentiviral vectors. Using the fine black precipitate indicative of PLAP enzymatic activity^10^,^16^, we detected infected mSCs using electron microscopy. We found no particular defect in the myelin ultrastructure of Crb3-silenced cells (Supplementary Fig. 4a). The myelin interperiodic distance and the g-ratio (axon diameter/fibre diameter) of Crb3-silenced cells were not different from non-infected cells (Supplementary Fig. 4b,c), confirming that Crb3 silencing did not significantly affect myelin thickness *in vivo*. Moreover we observed that labelled nodes of Ranvier, where the microvilli contacted the nodal membrane, did not appear disorganized (Supplementary Fig. 5a). Microvilli and node of Ranvier markers were correctly expressed and localized and the node length was unchanged by Crb3 silencing (Supplementary Fig. 5b,c), suggesting that the reduction of Crb3 expression did not affect microvilli or adjacent nodes of Ranvier. Because Crb3 silencing increases the myelin sheath length, without affecting the myelin thickness or the node of Ranvier, we conclude that Crb3 is an inhibitor of myelin sheath elongation *in vivo*.

### Hippo pathway and YAP mediate Crb3 effects

**Figure 2:**
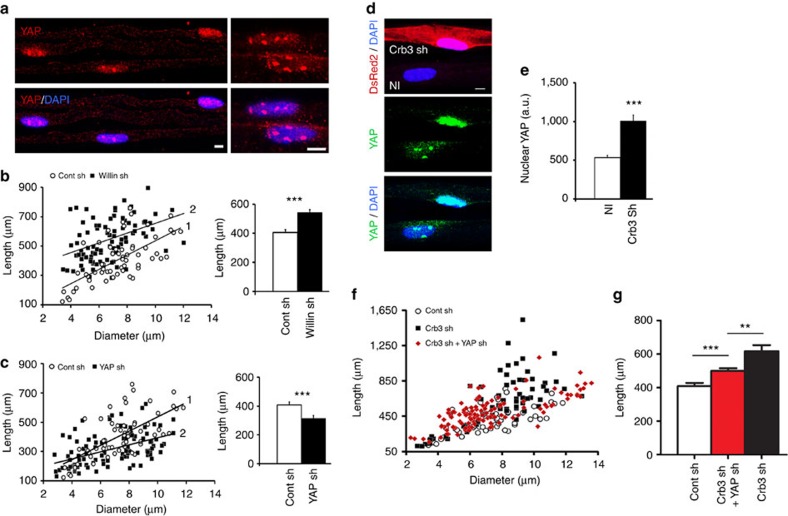
Nuclear YAP is regulated by Crb3 and the Hippo pathway. (**a**) YAP (red) is predominantly expressed in mSCs nuclei (DAPI, blue) in 15 dPN mice. Higher magnification in right panels. (**b**) Left: lengths versus diameters of mSCs infected with Willin (black square, *n*=88, slope line 2) or control (white circle, *n*=60, slope line 1) shRNAs lentiviruses. Two independent shRNAs against Willin yielded similar results (graphs show pooled data, three animals for each virus). Right: average length of mSCs described in left. (**c**) Left: lengths versus diameters of mSCs infected with YAP (black square, *n*=99, slope line 2) or control (white circle, *n*=60, slope line 1) shRNAs lentiviruses. Two independent shRNAs against YAP yielded similar results (graphs show pooled data, three animals for each virus). Right: average length of mSCs described in left. (**d**) More YAP (green) is expressed in the nucleus (DAPI, blue) of a cell expressing Crb3 shRNA (Crb3 sh, DsRed2) compared with a nearby non-infected cell (NI) at 60 dPN. (**e**) Quantification of YAP immunostaining in nuclei of Crb3-silenced mSCs (*n*=30 nuclei) versus surrounding non-infected mSCs (*n*=80 nuclei) at 15 dPN. Three animals for each virus. (**f**) New born mice mSCs were infected with control shRNA lentivirus (Cont sh, white circle) or with Crb3 shRNA lentivirus (Crb3 sh, black square) or with both Crb3 shRNA virus and YAP shRNA adenoviruses (Crb3 sh+YAP sh, red square). Previously published data^53^ have shown that co-injecting two adenoviruses resulted in co-infection in 97% of mSCs. Two months later infected cells were detected using fluorescent probes co-expressed with the shRNAs and measured. Co-infected cells have intermediate lengths compared to control and Crb3 shRNA virus infected cells. (**g**) Average length of mSCs described in **f**. Number of cells analysed: 59 (Cont sh), 118 (Crb3 sh+YAP sh), 65 (Crb3 sh). Three animals analysed for Control shRNA, four for Crb3 shRNA and three for YAP shRNA+Crb3 shRNA. AU: arbitrary units. Statistical two-tailed Student's *t*-tests were used. Scale bars, 5 μm (**a**,**d**). Error bars show s.e.m.

Crb3 can activate the Hippo pathway, via Merlin and/or Willin (also termed FRMD6)^17^,^18^, to prevent the shuttling of YAP transcription cofactor to the nucleus thereby inhibiting tissue growth due to nuclear YAP activity^19^ (Supplementary Fig. 6). We found that, in addition to Merlin^20^, Willin^18^, Mst1/2 and Lats1/2, and YAP were also expressed in the myelinated mouse peripheral nerve (Supplementary Fig. 7a). In immunostaining Merlin localized to Cajal's bands, while Willin localized to the Schwann cell microvilli (Supplementary Fig. 7b), suggesting that Willin but not Merlin is the target of Crb3 in mSC microvilli. In contrast YAP was mostly nuclear (Fig. 2a), while endogenous levels of Mst1/2 and Lats1/2 were too low to be detected by immunohistochemistry. As Willin is known to activate the Hippo pathway^17^,^18^, we therefore silenced Willin in Schwann cells *in vivo* using the viral approach described previously (Supplementary Fig. 7c,d). This silencing increased the myelin internodal length at two months compared with control shRNA (Fig. 2b), showing that Willin and the Hippo pathway participates in the control of myelin elongation. It is worth noting that Willin silencing affected more significantly thin fibres versus large fibres at the opposite to Crb3 (Supplementary Fig. 8), suggesting that additional molecules are involved. We then examined the consequence of silencing the final target of this pathway, YAP. Without YAP (Supplementary Fig. 7c,e), internodal length remained shorter than controls (Fig. 2c) and myelinated fibre diameter was also significantly thinner (6.6±0.23 versus 7.4±0.27 μm; *P*<0.05). In addition YAP silencing, such as Crb3 and Willin silencing, did not affect SC proliferation (Supplementary Fig. 7f) suggesting that silenced cells are not more abundant and the reduced internodal length is not due to competition for axonal space. So we conclude that YAP is required for optimal myelin length and diameter in mSC *in vivo*. To determine if Crb3 indeed controls YAP activity, we measured the amount of nuclear YAP in Crb3-silenced cells and found that these cells had more nuclear YAP (Fig. 2d,e) than neighbouring non-silenced cells. In addition, using co-infection with both Crb3 shRNA and YAP shRNA adenoviruses, we reduced YAP expression in the over-elongating Crb3-silenced cells. In this condition, mSCs over-elongated but significantly less than when Crb3 alone was silenced (Fig. 2f,g), showing that Crb3 required YAP to mediate to a full extent its effect on myelination. We conclude that Crb3 controls myelin sheath elongation via the Hippo pathway that negatively regulates YAP.

### YAP promotes myelin and non-myelin genes transcription

**Figure 3:**
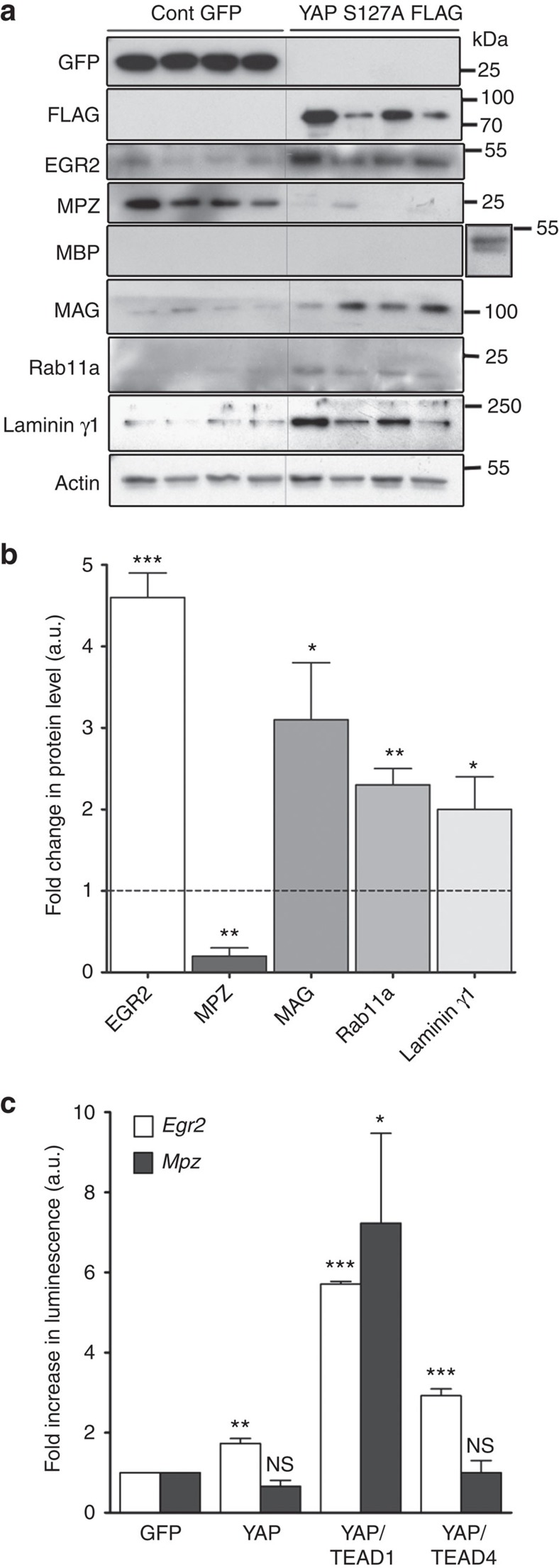
YAP directly regulates myelin and myelin-related protein expression (**a**) Western blots of differentiated primary rat Schwann cells following infection with GFP control or constitutively active YAP S127A Flag adenovirus. GFP and Flag panels confirm infection in four independent cultures of each condition. Myelin proteins EGR2 (∼50 kDa) expression is increased, MPZ (∼27 kDa) is downregulated and MBP (∼45 kDa) is unchanged by YAP activation. A positive control for MBP from whole sciatic nerve extract is shown in insert, right. MAG (∼100 kDa) is also upregulated as is expression of proteins with relevance to myelination, Rab11a (∼20 kDa) and Laminin1 (∼175 kDa), in response to YAP activation. Actin (∼48 kDa) was used to verify loading. (**b**) Quantification and statistical analysis of the change in selected protein expression in primary RSCs following differentiation and infection with dominant active YAP S127A, relative to levels in cells infected with GFP control (normalized to 1, dotted line). (**c**) HEK 293T cells were transfected with plasmids expressing *Mpz* or *Egr2* promoter driving Firefly luciferase expression, and either GFP, YAP alone or YAP and TEAD isoforms together. Luciferase levels were normalized to cells transfected with control GFP (three independent experiments in triplicates). Statistical analysis compares each transfection with the GFP infection for each promoter tested. a.u., arbitrary units. Statistical two-tailed Student's *t*-tests: *P<0.05; **P<0.01; ***P<0.001; NS, *P*>0.05 not significant (NS). Error bars show s.e.m.

YAP is a transcription cofactor that regulates gene expression through its binding to transcription factors including TEA domain factors, TEAD^21^,^22^. To understand how YAP regulates Schwann cell myelination we first investigated whether dominant active YAP S127A changes myelin protein expression in rat primary Schwann cells (RSCs) differentiated *in vitro*. Indeed early growth response protein 2 (EGR2), the key transcription factor required to promote myelination, and myelin-associated glycoprotein (MAG) were upregulated after YAP overactivation (Fig. 3a,b), while myelin protein zero (MPZ) was downregulated and myelin basic protein (MBP) remained undetectable. In addition, we found that YAP overactivation leads to the upregulation of Rab11 and Laminin γ1 expression (Fig. 3a,b), two proteins not specific to myelin but required for the myelination process^23^,^24^.

We then investigated if YAP can directly control myelin gene transcription at the promoter level focusing on MPZ, the main peripheral myelin protein, and EGR2, a key myelin gene regulator that controls MPZ expression^25^. Cells with either *Mpz* or *Egr2* promoters driving the expression of luciferase were transfected with control GFP or different YAP and TEAD constructs and the impact of these factors on luciferase expression was measured. YAP alone was able to stimulate the *Egr2* promoter but not the *Mpz* promoter (Fig. 3c). However, when cells co-expressed YAP and TEAD1, the *Mpz* promoter was strongly stimulated and the *Egr2* promoter activity was maximal. Co-expressing YAP with another TEAD family member, TEAD4, also stimulated the *Egr2* promoter but not *Mpz* (Fig. 3c), suggesting that effects of YAP on the *Egr2* and *Mpz* promoters partially rely on TEAD isoforms. We verified that TEAD isoforms are indeed expressed in the mouse sciatic nerve (Supplementary Fig. 9). Taken together these data confirm that YAP interacts with myelin gene promoters, eventually through TEAD factors, to control myelin elongation.

### Myelin elongation slows down when YAP is phosphorylated

**Figure 4:**
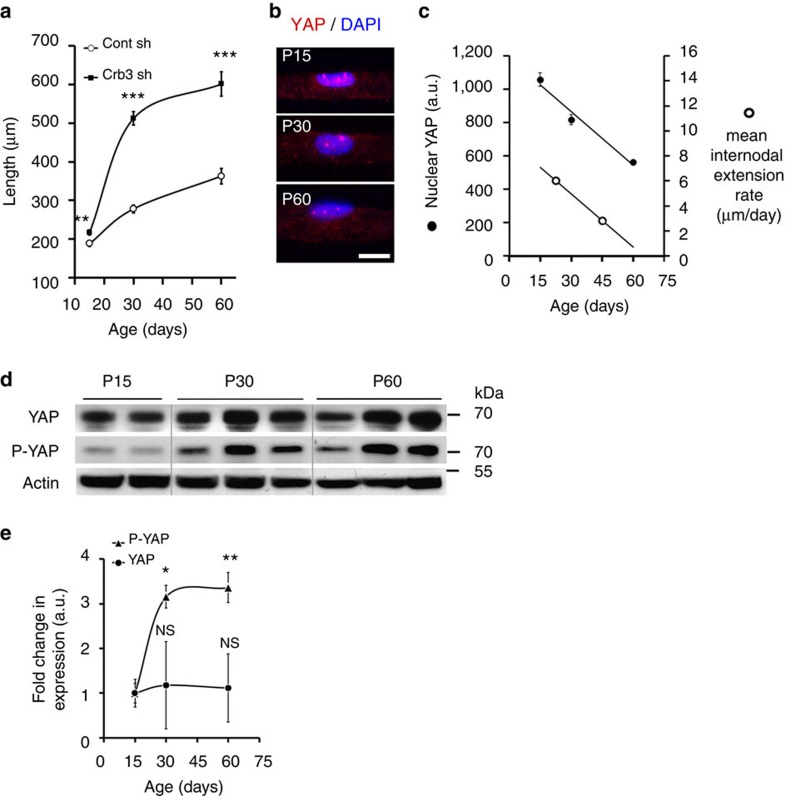
Developmental regulation of YAP during postnatal growth (**a**) The internodal lengths of mSCs infected with Crb3 (black square) or control (white circle) shRNA viruses injected at 3–5 dPN were measured at 15, 30 and 60dPN. Cells infected with Crb3 shRNA virus are significantly longer than cells infected with control ShRNA virus at all time points. Number of cell measured between 63 and 83, and three animals for each condition. Statistical analysis compares Crb3 sh with Cont sh at each time point. (**b**) Representative immunostaining of YAP immunostaining (red) in the mSC nucleus (DAPI, blue) during development at 15, 30 and 60 dPN (P). (**c**) Left axis: Quantification of nuclear YAP immunostaining (black circle) in mSCs at 15, 30 and 60 dPN. n between 62 and 164 cells from three animals at each age. Right axis: mean internodal extension rate at median time point 22.5 and 45 (white circle). Mean internodal extension (μm) was calculated between 15 and 30 dPN (median time point 22.5 dPN) and between 30 and 60 dPN (median time point 45 dPN) using the mean internodal lengths at 15, 30 and 60 dPN of control shRNA cells shown in **a**. The mean internodal extension rate is the ratio of mean internodal extension in μm over 15 (15–30 dPN) or 30 (30–60 dPN) days. (**d**) Representative western blots for YAP and P-YAP in mouse sciatic nerve at 15, 30 and 60 dPN (P). Each lane represents independent sciatic nerves. (**e**) Quantification of western blots for YAP and P-YAP at 15, 30 and 60 dPN (*n*=3, 4 and 4 independent experiments, respectively). Levels were first normalized for protein loading with actin, then the fold change in expression of individual replicates over time was expressed relative to the average level seen at 15 dPN. Statistical analysis compares YAP/Actin or P-YAP/Actin at 30 or 60 d to 15 dPN. Statistical two-tailed Student's *t*-tests: *P<0.05; **P<0.01; ***P<0.001; NS, *P*>0.05 not significant (NS). Scale bars, 10 μm (**b**). Error bars show s.e.m (**a**) and (**c**) and s.d. in **e**.

The rate of myelin sheath growth is not uniform during peripheral nerve myelination^3^,^26^,^27^, so obtaining a regular myelin sheath along a single axon requires a finely tuned developmental regulation. We therefore investigated whether Crb3 and YAP could participate in this process. Firstly, the effect of Crb3 silencing was analysed at different ages. At 15 dPN a small but significant change could be detected versus control shRNA (Fig. 4a). By 30 dPN the internodal length had more than doubled in Crb3-silenced cells (Fig. 4a), with a growth rate three times higher than in control cells (19.7 versus 6.0 μm per day). By 60 dPN, while the internodal length of Crb3-silenced cells remained higher than controls cells (Fig. 4a), both growth rates decreased to similar values (3 versus 2.8 μm per day). These data indicate that Crb3 silencing increases myelin elongation by boosting the growth rate without altering the timeframe of the elongation. As Crb3 is still significantly expressed in adult mouse nerve (Fig. 1a; Supplementary Fig. 1a) but its silencing no longer alters myelin internodal length (Supplementary Fig. 10a), this suggested that Crb3 inhibition of myelin elongation is not active at adult age. Consistently the expression of Crb3 target, YAP, in the nucleus of mSCs steadily decreased between 15 dPN and 60 dPN (Fig. 4b,c, left axis), paralleling the decrease of the mean internodal extension rate (Fig. 4c, right axis). This suggested that nuclear YAP amount is the limiting factor for myelin elongation. We then investigated the total levels of YAP and phosphorylated YAP, the inactive form generated by the Crb3/Hippo pathway, in sciatic nerve by western blot. We found that while total YAP levels remained constant between 15 and 60 dPN, the level of phospho-YAP strongly increased after 30 dPN (Fig. 4d,e), indicating that the Crb3/Hippo pathway controls the amount of active nuclear YAP during myelination. Taken together our data confirms that the negative developmental regulation of YAP by the Crb3/Hippo pathway coincides with and explains the decreasing rate of myelination as internodes reach their required final length.

### Nerve stretching triggers myelin elongation via nuclear YAP

**Figure 5:**
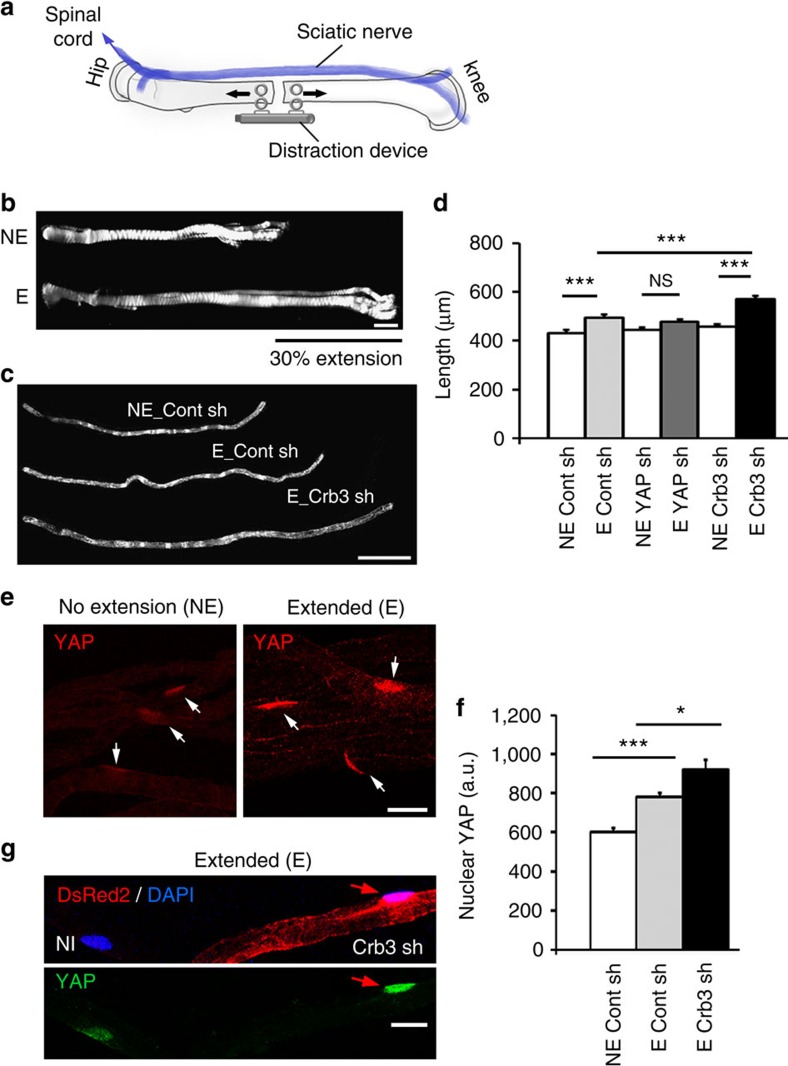
Nuclear expression of YAP is regulated by nerve elongation (**a**) Schematic of the technique we used to mechanically distract the adult (11 to 13 weeks old) mouse femur and to artificially extend the sciatic nerve (blue) that is attached to the spinal cord (device not to scale, see Online Methods for more details). (**b**) An entire extended sciatic nerve (E) and the non-extended contralateral nerve (NE) of the same mouse are shown after femoral distraction using reflected white light. The extended sciatic nerve is 30% longer than the contralateral nerve. (**c**) Representative mSCs infected with control (Cont sh) or Crb3 (Crb3 sh) shRNA virus in an extended (E) or a non-extended nerve (NE). (**d**) Quantification of internodal lengths of cells infected with control, YAP shRNA or Crb3 shRNA viruses in extended (E) or non-extended nerves (NE). Between 106 and 171 cells (from three animals) were measured for each condition. (**e**) Nuclear YAP (red) immunostaining in mSC nuclei (arrows) is higher in artificially extended nerves (E, right panel) than in non-extended nerves (NE, left panel). (**f**) Quantification of YAP immunostaining in nuclei of mSCs infected with control or Crb3 shRNA viruses in extended (E) or non-extended nerves (NE). Nuclei quantified: 52 (NE Cont sh), 131 (E Cont sh), 19 (E Crb3 sh). Three animals for each condition. (**g**) In an extended nerve, the nucleus (arrows) of a cell expressing Crb3 shRNA (red) has more YAP (green) than the nucleus (DAPI, blue) of a nearby non-infected cell (NI). a.u., arbitrary unit. Statistical two-tailed Student's *t*-tests: *P<0.05; **P<0.01; ***P<0.001. Scale bars, 1 mm (**b**), 100 μm (**c**), 20 μm (**e**,**g**). Error bars show s.e.m.

**Figure 6:**
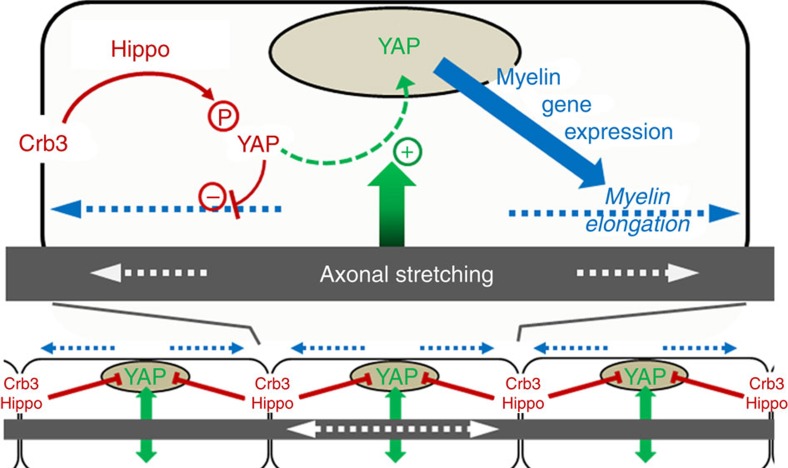
Schematic illustrating the model of myelin elongation in mSC. Top: A myelinating Schwann cell is exposed to input (green arrow) from the stretching axon in the elongating nerve which stimulates the translocation of YAP to the nucleus (green dotted line). YAP nuclear activity promotes myelin gene expression (blue arrow), which increases myelination and myelin elongation (blue dotted arrows). YAP nuclear translocation is negatively regulated by Crb3/Hippo pathway signalling originating from the cells extremities (long red arrow) which phosphorylates YAP, sequestering it in the cytoplasm which consequently limits myelin elongation (short red line). Bottom: the combination of signals promoting YAP activity (green arrows) and inhibiting it (red lines) allows the harmonious growth (blue dotted lines) of all myelin segments along the axon.

We next examined what initially drives YAP activation and localization to the nucleus. As internodal length strongly correlates with nerve and limb elongation^27^ we hypothesized that axonal elongation, which occurs by stretching with postnatal body growth *in vivo*^28^, is required to drive YAP nuclear localization and myelin elongation. To test this hypothesis we used a femoral distraction technique to mechanically elongate the sciatic nerve of adult mice (Fig. 5a). This technique enabled nerve extension by ∼30% over 15 days in living mice (Fig. 5b), without damaging axons^29^. As previously observed in rat and rabbit^29^,^30^, nerve stretching in adults steadily stimulated myelin sheath elongation (Fig. 5c,d, two first columns). This correlated with increased YAP expression in the nucleus of myelinating cells (Fig. 5e,f). When cells were silenced for YAP myelin did not elongate following nerve extension (Fig. 5d, two middle columns). In addition we observed that these YAP-silenced cells had thinner extremities and showed a heterogeneous structure (Supplementary Fig. 10b,c). This suggested that the loss of YAP under elongation conditions prevents the mSC from ‘keeping up' with the imposed artificial extension. While Crb3 loss has no effect on the internodal length of mature mSCs (Supplementary Fig. 10a), the re-engagement of the Crb3/Hippo/YAP pathway in artificially extended adult nerves, allowed Crb3 silencing to increase nuclear YAP (Fig. 5f,g) and myelin elongation (Fig. 5c,d, two last columns) at even higher levels than in control cells of similarly extended nerves. This indicated that Crb3 can still play an inhibitory role on myelin elongation in adult nerves when axonal stretching stimulates YAP nuclear activity. We conclude that myelin elongation during development is initiated by the postnatal limb elongation and axonal stretching that recruits YAP to mSC nucleus; it is then negatively controlled by Crb3, via the Hippo pathway, to allow harmonious growth of all myelin segments along the axon (Fig. 6).

### Crb3-Hippo dissociates longitudinal from radial growth

**Figure 7:**
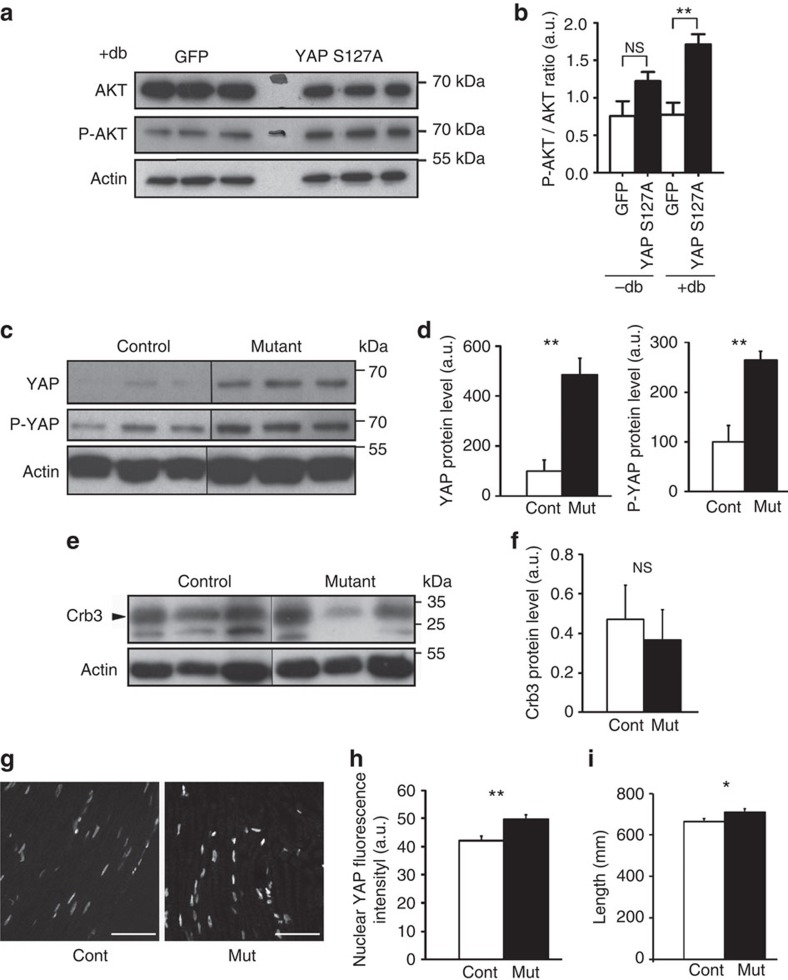
Crb3-Hippo pathway dissociates longitudinal from radial myelin growth. (**a**) Western blots showing expression of AKT and active phosphorylated AKT (P-AKT) in primary rat Schwann cells differentiated in culture with dibutyryl-cyclic AMP (+db) following infection with GFP control or constitutively active YAP S127A Flag adenovirus. GFP and YAPS127A panels confirm infection in three independent cultures of each condition. Actin is used as loading control. (**b**) Ratio active P-AKT on AKT is calculated from the quantifications of the western blots shown in **a** and after normalization over actin. Three independent experiments. NS: not significant. (**c**) Representative western blots showing expressions of YAP and phosphorylated YAP (P-YAP) in sciatic nerves of transgenic Nrg1 mice (Mutant) and of control littermate. Actin is used as loading control. (**d**) Quantifications of western blots showing YAP and phosphorylated YAP levels in mutant mice (Mut, 5 mice) relative to control mice (Cont, 4 mice) normalized over actin levels. (**e**) Representative western blots showing expression of Crb3 in sciatic nerves of transgenic Nrg1 mice (Mutant) and of control littermate. Actin is used as loading control. (**f**) Quantifications of western blots showing Crb3 levels in mutant mice (Mut, 5 mice) relative to control mice (Cont, 4 mice) normalized over actin levels. Error bars show s.d. (**g**) Representative nuclear YAP immunostaining on sciatic nerve longitudinal sections of transgenic Nrg1 mice (Mut) and littermate control (Cont). Scale bar, 50 μm. (**h**) Quantification of YAP immunostaining fluorescence intensity in nuclei of mSCs as shown in **e**. Nuclei quantified: 112 (Control), 270 (Mutant). Three animals for each condition. (**i**) Quantification of internodal lengths after teasing sciatic nerves of transgenic mice (Mut, *n*=57 cells, three animals) and control littermate (Cont, *n*=84 cells, three animals). Cells were stained with FluoroMyelin, nuclei with TOPRO3 and nodes immunostained with Kv1.2 antibodies. Mice were 5 months old. a.u., arbitrary units. Statistical two-tailed Student's *t*-tests: *P<0.05; **P<0.01; NS: not significant. Error bars show s.d for **b**, **d** and **f** and s.e.m for **h** and **i**.

Axonal neuregulin 1 type III (Nrg1) has been shown to be essential to control myelin growth. Indeed Nrg1 overexpression in axons leads to an abnormal thickening of the myelin sheath due to the overactivation of the PI3K-AKT pathway^12^,^11^ but no change in the internodal length^31^. On the opposite Crb3 silencing affected myelin length but not diameter and YAP silencing affected both parameters. To understand the interplay between Crb3-Hippo-YAP and Nrg1-PI3K-AKT pathways we first expressed constitutively active YAP in differentiated SC in presence of soluble Nrg1. YAP reduced AKT expression but strongly promoted its phosphorylation (Fig. 7a,b), indicating that YAP promotes AKT activity in SC. We then collected sciatic nerves of transgenic mice overexpressing Nrg1 in neurons^11^ and compared YAP and phospho-YAP expressions with littermate controls. Mutant mice expressed more YAP but also more phospho-YAP (Fig. 7c,d). As Crb3 expression did not change (Fig. 7e,f), this suggested that the Crb3-Hippo pathway was efficiently inactivating the excess of YAP. As a result, active nuclear YAP increased only slightly in SC of transgenic mice (Fig. 7g,h). Finally, internodal length increased also in mutant mice but this increase remained small and nearly non-significant (Fig. 7i; Student *t*-test *P* value=0.035). Taken together these data indicates that axonal Nrg1 signalling upregulates YAP but this increase does not affect much the internodal length because YAP is largely inactivated by the Crb3-Hippo pathway.

### Lack of nuclear YAP leads to shorter myelin in *Dy* ^*2j/2j*^ mice

**Figure 8:**
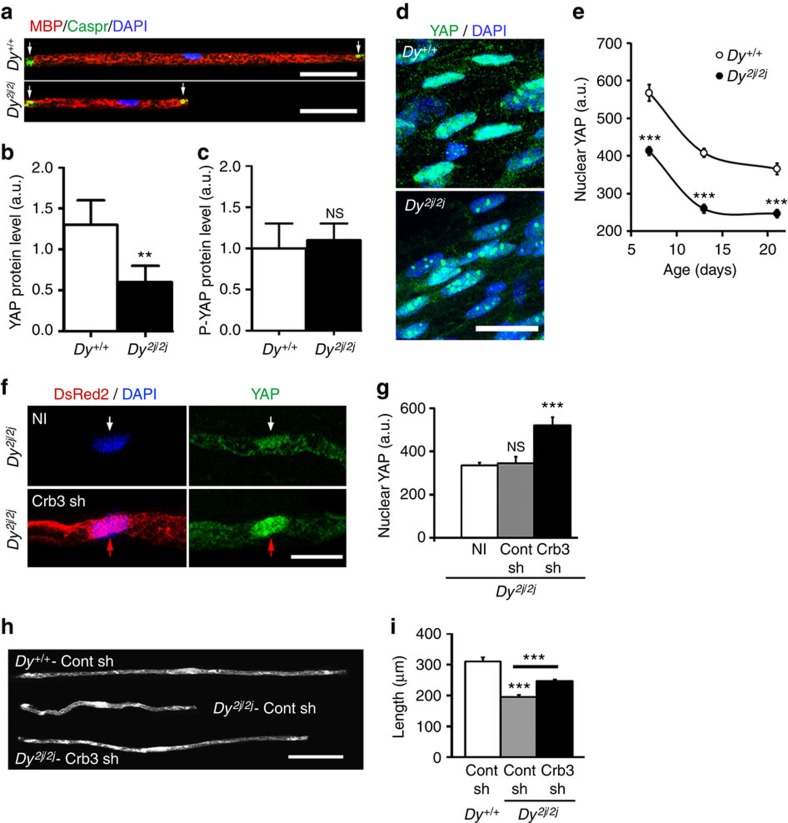
Nuclear YAP is reduced in mSCs of *Dy*^*2j/2j*^ mice and increasing nuclear YAP rescues short internode phenotype. (**a**) Teased nerve fibres of *Dy*^*2j/2j*^ mice or wild-type littermates (*Dy*^*+/+*^) at 15 dPN immunostained for myelin (MBP, red), paranodes (Caspr, green) and nuclei (DAPI, blue). (**b**,**c**) Quantification of total YAP (**c**) and phosphorylated YAP (P-YAP) (**b**), normalized to actin, in sciatic nerves from *Dy*^*+/+*^ (4 animals) and *Dy*^*2j/2j*^ (five animals) littermates. (**d**) YAP immunostaining (green) in mSCs nuclei (DAPI,blue) at 7 dPN in *Dy*^*2j/2j*^ and *Dy*^*+/+*^ littermates. (**e**) Quantification of YAP immunostaining in nuclei of *Dy*^*2j/2*j^ (black circle) and *Dy*^*+/+*^ mSCs (white circle) at 7, 14 and 21 dPN. The number of nuclei analysed between 38 and 120 (three animals). (**f**) A *Dy*^*2j/2j*^ mSC infected with Crb3 shRNA virus (lower panels, DsRed2) has more YAP (green) in its nucleus (DAPI, blue, red arrows) than a non-infected (NI) cell in the same nerve (upper panels, white arrows). Virus injection at 3–4 dPN and sacrifice at 30 dPN. (**g**) Quantification of nuclear YAP immunostaining in *Dy*^*2j/2j*^ mSCs, not-infected (NI, 87 nuclei) or infected with control (Contsh, 18 nuclei) or Crb3 (Crb3sh, 13 nuclei) shRNA viruses. (**h**) mSCs infected with Control shRNA virus in a control littermate nerve (*Dy*^*+/+*^ Cont sh, GFP, top) or in a *Dy*^*2j/2j*^ mutant mouse nerve (*Dy*^*2j/2j*^-Cont sh, GFP, middle) or infected with Crb3 shRNA virus in a *Dy*^*2j/2j*^ mutant mouse nerve (*Dy*^*2j/2j*^-Crb3 sh, DsRed2, bottom). Mouse pups were injected at 3–4 dPN and sacrificed at 30 dPN. (**i**) Quantification of internodal lengths of cells described in **h**. The reduction of internodal length in cells of *Dy*^*2j/2j*^ mice (*Dy*^*2j/2j*^-Cont sh, 41 cells) versus wild-type littermates (*Dy*^*+/+*^ Cont sh, 62 cells) is partially compensated when *Dy*^*2j/2j*^ mouse cells are silenced for Crb3 (*Dy*^*2j/2j*^-Crb3 sh, 76 cells). a.u., arbitrary units. Scale bars, 50 μm (**a**), 15 μm (**d**), 20 μm (**f**) and 100 μm (**h**). Statistical two-tailed Student's *t*-tests: *P<0.05; **P<0.01; ***P<0.001. Error bars show s.d in **b**, **c** and s.e.m in **e**, **g** and **i**.

Reduced internodal length strongly impairs nerve conduction velocity^2^,^4^ and some human peripheral neuropathies have been shown to be linked to reduced internodal length such as CMT4F (*Periaxin* gene mutations)^6^,^7^,^8^, and congenital muscular dystrophy 1A (*α2 laminin* gene mutations)^9^. To check whether the Crb3/Hippo/YAP mechanism is involved in these diseases we investigated a naturally occurring mouse line, *Dy*^*2j/2j*^, mutated on α2 laminin gene that displays a peripheral neuropathy with reduced internodal length^32^ (Fig. 8a) mimicking the human congenital neuro-muscular dystrophy 1A (ref. 9). We found that mutant mice sciatic nerves had less YAP (Fig. 8b) than control littermates but no change in phospho-YAP or Crb3 expression (Fig. 8c; Supplementary Fig. 11). Moreover mutant mSCs had significantly less nuclear YAP (Fig. 8d,e). Taken together this suggested that decreased nuclear YAP may be responsible for the reduced internodal length. To confirm this we sought to restore nuclear YAP level in mSCs of *Dy*^*2j/2j*^ mice. Because YAP overexpression was deleterious and lifting the Hippo pathway inhibition on YAP via Crb3 silencing increased nuclear YAP amount in wild-type mice (Fig. 2d,e), we silenced Crb3 in mutant mSCs. As expected this increased the amount of nuclear YAP (Fig. 8f,g) and accordingly increased the internodal length of mutant cells (Fig. 8h,i), confirming that the lack of nuclear YAP contributes to the peripheral nerve defect in these mice. Our data therefore suggest that peripheral neuropathies with reduced internodal length are due to defects in the expression of YAP in the Schwann cell nucleus and it may be possible to correct suboptimal internodal lengths by artificially promoting YAP activity via manipulation of Crb3.

## Discussion

We found that myelin elongation in mSCs is driven by the stimulation of YAP transcription cofactor activity during the axonal elongation of postnatal body growth. The localization of YAP in nucleus, and therefore its ability to promote myelin gene transcription, is regulated by Crb3 and Willin, upstream controllers of the Hippo pathway, as evidenced by the accumulation of phospho-YAP coincident with decreased nuclear YAP at 30–60 dPN during the slowing of myelin extension. Thus YAP nuclear activity is stimulated by nerve elongation during body growth and conversely tempered by Crb3 signalling originating at the longitudinal extremities of the mSC. Taking these opposing forces into account we propose a model that accounts for optimal and homogeneous longitudinal growth of adjacent myelin segments (Fig. 6).

The Nrg1-PI3K-AKT pathway has been shown to be critical during myelination and in particular to control the myelin geometry^10^,^11^,^12^. However manipulations of this pathway revealed that other mechanisms were likely to be involved in the promotion of myelination in Schwann cells and in oligodendrocytes, the myelinating glia of the central nervous system^33^,^34^. The YAP pathway may represent one of these supplementary mechanisms. Indeed YAP silencing reduced myelination and increasing YAP activity via Crb3 silencing increased longitudinal myelination.

How YAP, a co-transcription factor, stimulates myelination is not completely clear but our data gives some indications. First, YAP activation in cultured SC positively (EGR2, MAG) or negatively (MPZ) regulates myelin protein expression and it controls myelin gene transcription at the promoter level. This is nevertheless not limited to myelin genes as YAP also promotes expression of non-myelin proteins that are critical for myelination such as Rab11 or Laminin γ1. However YAP does not act alone and our data suggest that its functional interactions with TEAD transcription factors to promote myelin gene transcription are complex. So, additional studies will be required to fully understand the role of YAP on gene transcription in mSC.

Second, YAP can promote myelination via the Nrg1-PI3K-AKT pathway. Indeed constitutively active YAP strongly increases AKT activation in differentiated SC in culture in presence of Nrg1. It has been reported that YAP promote the expression of the catalytic subunit of PI3K, p110γ, which activates AKT^35^, but whether this is occurring in mSC remains to be shown. In any case Nrg1 and YAP pathways strongly interact in mSC. Indeed we found that Nrg1 overexpression in neurons, which increases myelination, strongly stimulates YAP expression. How this occurs is unclear but this is consistent with our finding that nuclear YAP expression is promoted by axonal clues during development.

However the most intriguing part is the dissociation between the longitudinal and the radial growth of myelin. Indeed while during development myelin diameter and length increases harmoniously and are proportionate to the axon diameter, it has been shown that internodal length is actually more significantly related to the nerve length^27^. This dissociation is reflected by human peripheral nerve diseases as some of them show short myelin segment with a correct diameter^9^. In addition, overexpression of axonal Nrg1 (refs 11, 31) and activation of the PI3K-AKT pathway^10^ in mSC increase radial but not longitudinal myelin growth. On the reverse Crb3 silencing and artificial axonal elongation promote only longitudinal myelin growth. Using mice overexpressing axonal Nrg1, we found that while YAP is upregulated the Crb3-Hippo pathway phosphorylates and inactivates it. So nuclear YAP does not increase much and myelin longitudinal growth remains close to normal, while the upregulation of the PI3K-AKT pathway increases radial growth. This suggests that the dissociation results from the inhibition of YAP activity via Crb3 and the Hippo pathway.

It has been reported that AKT can phosphorylate and inactivate YAP^36^, which could also explain why the majority of YAP is phosphorylated in Nrg1 overexpressing mutant. However if AKT was a major regulator of YAP phosphorylation then nuclear YAP should be much lower in growing pups because at that age AKT activity is high^10^. In addition when we silenced AKT1 or AKT2 in mSC we obtained shorter myelin^10^, which is not consistent with lower YAP phosphorylation. So we believe that AKT is not likely to the main regulator of YAP activity in mSC and this role is essentially played by the Crb3-Hippo pathway.

It remains unclear how Crb3-silenced mSCs can elongate their myelin sheath without making it larger, while during the same developmental timeframe Dlg1-silenced cells enlarge it without making it longer^10^. This suggests that, beyond the production of myelin and interactions between YAP and Nrg1 pathways, Crb3 and YAP are involved in mechanisms that control the longitudinal deposition of myelin. We propose that multiple local points of myelin synthesis exist along the myelin sheath, most likely at the outside edge of incisures and of paranodal loops (Supplementary Fig. 2e), with local enrichment in phospho-AKT and eventually mTOR complexes. Crb3 and YAP could promote the formation of these myelinogenesis points and therefore promote myelin elongation. In a second mechanism YAP could also promote the trafficking along the myelin sheath and therefore favour the longitudinal deposition of myelin. We envision these two mechanisms may co-exist and speculate that while radial myelination could be due to more myelin production only, longitudinal myelination may be due to both more myelin production and more myelinogenesis points along the internode. It would be interesting to examine the consequence of relieving Crb3 inhibition while increasing myelination via the Nrg1-AKT pathway. In this case the excess of myelin might be deposited longitudinally instead of making the myelin thicker. Finally it can be noted that both longitudinal and radial myelination pathways are finely regulated by negative signalling coming from the stabilization of Dlg1 and PTEN proteins^10^ and from the Crb3/Hippo pathway (this study), respectively. This combination of positive and negative conflicting regulations may be the golden key for the optimization of the myelin sheath geometry in PNS and CNS.

Our data suggest that Crb3 act as a sensor in the microvilli for signals coming from neighbouring cells or from the axonal node of Ranvier (Supplementary Fig. 2e). Activated by these signals Crb3 could activate Willin and the Hippo pathway, phosphorylate and prevent YAP access to the nucleus and thereby inhibit myelination. Crb3 features a short extracellular domain which may interact with the extracellular matrix or with other proteins extracellular domains in cis or in trans^37^. Another possibility could be that Crb3 interacts with its transmembrane domain or its intracellular domain with other transmembrane proteins that will act as the sensor protein for extracellular signals. Willin was first detected in peripheral nerve tissues as an interacting protein for Neurofascin using two-hybrid technique^38^ and Neurofascin 155 (NF155) is expressed in some conditions in the SC microvilli^39^; so it is possible that Crb3 is engaged in a complex with Willin and NF155 in mSC microvilli. NF155 may therefore be the transmembrane protein responsible for sensing the extracellular signals and for translating it into mSC via Crb3 and the Hippo pathway. However whether the loss or the inactivation of NF155 in mSCs results in longer internodal length remains undocumented. As Crb3 and Willin silencing do not affect large and thin fibres the same way, other molecules such as Crb1/Crb2, or nectins^40^,^41^ may also be involved in the signal transduction. However the link of these molecules with the cell polarity is less clear. So, additional work is required to discover the complete mechanism that is engaged during the control of longitudinal myelination in peripheral nerves.

We found that in adult nerve relieving Crb3 inhibition on myelin elongation does not lead to longer cells, despite the continued expression of Crb3. To see the inhibitory effect of Crb3 in adult mSCs we have to re-engage axonal extension by artificially extending the nerve. This means that Crb3 is not functionally inactivated in adult mice but that its activity is silent because most of the YAP is already phosphorylated and inactive. When we re-engage axonal extension more unphosphorylated YAP is generated so the Crb3 activity can be seen again. This is supported by the strong YAP phosphorylation that occurs in adult transgenic Nrg1 mice as it suggests that Crb3 and the Hippo pathway are still very active in adult mice. This is also consistent with the proposal that Crb3 is activated by cell–cell interaction at the microvilli, because this does not stop at adult age.

A major implication of our data is that nerve extension promotes nuclear YAP localization and stimulates myelin sheath elongation. These data are consistent with empirical observations showing that myelin internodal length correlates with the limb growth during development^27^. Nevertheless, the molecular mechanisms that underlie this process remain unclear. When nerves are extended axons are also stretched and elongate by inserting new material all along their length^42^. This elongation mechanism is different from the axonal growth that occurs during embryonic development and *in vitro*, which is mediated by the axonal growth cone. Our data suggest that the extending axons send a signal to the mSC. This signal might originate from axo-glial junctions that link glial paranodal loops to paranodes of the node of Ranvier (Supplementary Fig. 2e). As it is lying directly on the axon, one way that Schwann cell has to detect axonal stretching is to anchor both glial extremities on the stretched axon and to use the glial cytoskeleton as a tension sensor. But this may not be the only one.

Indeed physical interactions between the glial abaxonal plasma membrane, its underlying cytoskeleton and the extracellular matrix could also play a critical role in the detection of axonal stretching. The abaxonal membrane (Supplementary Fig. 2e) contains dystroglycans that interact with the laminin of the basal lamina. These dystroglycans interact with Periaxin and Utrophin, which are linked to the cytoskeleton^3^,^43^,^44^. Mutations in *periaxin* gene lead to Charcot-Marie-Tooth disease 4F, a peripheral neuropathy with reduced internodal length^3^, as observed in congenital muscular dystrophy patients with mutations in the *laminin-2* gene^45^. In addition mouse mutants for dystroglycans and Utrophin display a reduced internodal length^43^, showing that the interaction between the basal lamina and the glial cytoskeleton is required for myelin elongation. We therefore suggest that the anchoring of the Schwann cell to the basal lamina via dystroglycans and the related cytoskeleton participates in the detection/sensing of axonal stretching by offering a stable reference to oppose to the extending axon.

Highlighting the role of the Crb3/Hippo/YAP pathway in the emerging picture of myelin formation confirms that this process is far from passive. It is highly controlled in space and time by a combination of positive and negative signalling and by the superposition of different signalling pathways^46^,^47^. This growing complexity will give us the opportunity to build a detailed *in silico* cellular model in which we will be able to identify molecular targets for drug or gene therapies for peripheral neuropathies.

*Note added in proof:* a recent publication describes the role of YAP in axonal sorting by Schwann cells in the mouse peripheral nerve^58^.

## Methods

### Mice

Pregnant (E15-16) and adult Swiss mice were purchased from Janvier (France). Adult CB17/SCID mice were purchased from Charles River Laboratories (France) and CB17/SCID pups were obtained from breeding in our animal facility. Sciatic nerves of transgenic Nrg1 (Thy1.2-Nrg1 type III; 5 months old) mice and control littermate were a kind gift of R. Fledrich and R. Stassart (Department of Neurogenetics, Max Planck Institute of Experimental Medicine, Göttingen, Germany). Heterozygous Lama2 *Dy*^*+/2j*^ mice were purchased from The Jackson Laboratory (USA) and used to generate *Dy*^*+/+*^ and *Dy*^*2j/2j*^ mice for experiments. As a point mutation in the *Dy*^*2j*^ allele contains a unique Nde1 restriction site, we used a genotyping strategy to PCR across the point mutation followed by Nde1 restriction digestion of PCR products as previously described^48^. Briefly DNA was extracted from ear biopsies and PCR performed with Phusion HF Taq polymerase (New England Biolabs, France) using the following sense (-S) and antisense (-AS) primers: S: 5- TCC TGC TGT CCT GAA TCT TG -3; AS: 5- CTC TAT TAC TGA ACT TTG GAT G -3. PCR products were digested overnight with Nde1 (New England Biolabs, France) to produce an uncut 377 bp product in wild type and digested 164- and 109-bp fragments from *Dy*^*2j/2j*^ mice. Heterozygous mice were identified by the presence of all three bands.

### Animal surgery and injection

Animal use was approved by the Comité Régional d'Ethique du Languedoc –Roussillon, France and the veterinary office of the Canton of Zurich, Switzerland. Injection of viral constructs in pup and adult mice were conducted as described previously^15^,^49^. Briefly, postnatal day 3–4 mice (as indicated) or postnatal day 30 CB17/SCID mice were anaesthetized with isoflurane inhalation and placed under a Zeiss stereomicroscope (Stemi 2000, Carl Zeiss Microscopy GmbH). The gluteus superficialis and biceps femoris muscles were separated to reveal the cavity traversed by the sciatic nerve. A thin glass needle filled with coloured viral solution (3 μl for pups or 8 μl for adult injections) was introduced into the nerve with a micromanipulator (IM—3C, Narishige Japan Group). This solution was injected over 10 min with short pressure pulses using a microinjector (Pneumatic Picopump PV820, World Precision Instruments) coupled to a 3 MHz function pulse generator (GFG8215, Langlois), so that regions distant of the injection site could be filled. The nerve was replaced in the cavity, the muscles were readjusted, and the wound was closed with Histoacryl glue (BBraun, Germany). When fully awake, pups were put back to the mother and the litter treated with antibiotics to prevent infection. Sacrifices were done by CO_2_ inhalation.

### Lentivirus production and purification

One 15 cm polylysine-coated dishes of 95% confluent HEK 293T cells was transfected with 13.5 μg pSPAX2, 9 μg pMD2G and 27 μg lentiviral vector. The supernatant was collected 48 and 72 h later, pooled, centrifuged at 1,000 r.p.m. and filtered. The supernatant was centrifuged using the SW28 rotor, 21,000 r.p.m. at 11 °C (Beckmann Coulter) for 2 h. The resulting pellet was re-suspended in 1 ml DMEM 10% FCS without antibiotics and centrifuged again for 1 h at 16,000 r.p.m. and 4 °C using the T60i rotor (Beckmann Coulter). The pellet obtained was finally re-suspended in 40 μl PBS with 0.001% fast green (Sigma), and aliquots stored at −80 °C. Titres were between 10^10^ and 10^11^ TU ml^−1^. These viral preparations were used to infect mSCs *in vivo*.

### Adenovirus production and purification

Adenoviruses were produced from the pAdeasy adenoviral vector system by Sirion Biotech (Martinsried, Germany). Titres were between 10^11^ and 10^12^ TU ml^−1^. They were used in both the *in vivo* infection of mSCs and for infection of myelinating co-cultures.

### ShRNAs, plasmids and viral vectors construction

The lentiviral pLKO.1 vectors containing shRNA targeting mouse Crb3:

1- #TRCN0000124547, 5′ CTTGGCGTTTGGTTTGCCGAT ;

2- #TRCN0000124548, 5′ GCCTAACAGCACCGGACCCT T;

3- #TRCN0000124545, 5′ CGGACCCTTTCACAAATAGCA ;

4- #TRCN0000124544, 5′ CCAACAAACCTCCTGTCCTTT ; are from SIGMA. These vectors express puromycin and were used for low titre lentiviral production and shRNA validation in mouse EpH4-J3B1A cell line.

The Crb3 shRNA sequences 3 and 4 described above, the validated mouse shRNA sequences for YAP (shRNA1 5′ CGGTTGAAACAACAGGAATTA ; shRNA2 5′ GCCACCAAGCTAGATAAAGAA )^50^,^51^ and Willin (shRNA1 5′ GACTCGCAAGATGAGGAAAT ; shRNA2 5′ GACCTTACTCACGACGAAGT )^52^ were cloned into the lentiviral pSICOR (Addgene 11579) vector using HpaI-XhoI sites^16^. These vectors were used for high titre lentivirus production and *in vivo* injection. In addition Crb3 shRNA 3 and 4, and YAP shRNA 1 sequences were cloned in the adenoviral pAdt shRNA plasmid as described previously^53^. Lentiviral and adenoviral vectors gave similar results.

Crb3 shRNA sequences 3 and 4 were also cloned using the restriction sites described above in pSICOR vector where GFP was replaced with PLAP as described previously^10^.

Control shRNAs were directed against DsRed2 (5′ AGTTCCAGTACGGCTCCAA ) or GFP (5′ CAAGCTGACCCTGAAGTTC ) depending on the fluorescent reporter used (pSICOR expresses GFP while the pAdt shRNA expressed DsRed2)^53^. Both control shRNAs gave similar results and had no effect on myelin^16^,^53^. SIGMA control shRNA (#SHC002) was also used for retro and lentiviral vectors. Control virus expressing GFP alone were either lentiviral pSICOR with no shRNA or adenoviral pAdTrackCMV expressing GFP alone.

As both Crb3 shRNAs (3 and 4), Willin shRNAs (1 and 2), or YAP shRNAs (1 and 2) gave similar effects on myelin elongation between P3 and 2mPN (Figs 1e,f and 2b,c), we used Crb3 shRNA3, Willin shRNA1 and YAP shRNA1 in the following experiments.

### Electron microscopy and PLAP staining

Nerves infected with adenoviral vectors expressing Crb3 shRNA 3 or 4 and PLAP were fixed and processed for electron microscopy as described previously^10^. Samples were analysed with a HITACHI H 7100 transmission electron microscope at the Centre Regional d'Imagerie Cellulaire, Montpellier, France. Statistical analysis was done with four samples of nerves infected with each virus. For each cell showing a PLAP staining and for 2–4 non-labelled surrounding cells, axonal and myelin perimeters were measured using ImageJ software to obtain their diameters. From these values, the g-ratio of each cell (axon diameter/myelin diameter) was calculated.

### Teased fibres preparation and staining

Teasing of sciatic nerves has been published previously^10^. Briefly, sciatic nerves were isolated from injected mice, fixed for 10 min in Zamboni's fixative, washed in PBS and subsequently teased on glass slides. After teasing the nerves were dried overnight at room temperature and immunostaining was performed. For immunostaining, the teased fibres were first incubated for 1 h at room temperature in blocking solution (5% goat serum, 5% donkey serum, 0.3% Triton X-100 and 0.01% sodium azide in PBS), then incubated with primary antibodies in blocking solution overnight at 4 °C. Slides were washed in PBS and incubated for 1 h at room temperature with secondary antibodies diluted in the blocking solution. Finally, samples were washed in PBS and mounted in ProLong Gold Antifade Reagent with DAPI (Molecular Probes). Images were acquired at room temperature using a 20 × , 40 × or 63 × C-Apochromat objectives on a Leica confocal microscope SP5 or SP8. Pictures were then saved in TIFF format and processed with Image J and compiled into figures in Photoshop (Adobe).

### Antibodies for immunostaining

All primary antibodies used are listed as follows:

Rabbit anti-Crb3 (D2, kind gift from Dr A.Le Bivic, IBDML, France, 1:200)

Mouse anti-Moesin (610401, BD transduction, 1:100)

Mouse anti-AnkG (NB20, Calbiochem, 1:50)

Rabbit anti-Gliomedin (KR34, Koch, 1:1,000)

Mouse anti-KV1.2 (75-008, Antibodies incorporated 1:500)

Rabbit anti-pERM (3149, Cell Signaling, 1:100)

Rabbit anti-pan-Neurofascin (563, kind gift of Dr L. Goutebroze, Institut du Fer à Moulin, Paris, France, 1:1,000)

Rabbit anti-Nav1.8 (AP672-4.1, kind gift of SR Levinson, university of Colorado Health Center, USA, 1:200)

Rabbit anti- Caspr/paranodin (L51, kind gift from Dr L. Goutebroze, Institut du Fer à Moulin, Paris, France, 1:1,000)

Mouse anti-MBP (SMI94, Sternberger Monoclonal incorporated, 1:10,000)

Rabbit anti-Willin (Ex988-1, kind gift from M. Takeichi, RIKEN Center for Developmental Biology, Japan, 1:200)

Rabbit anti- YAP (4912, Cell Signaling, 1:200)

Secondary antibodies for teased fibres immunostaining were used at 1:1,000 dilutions. To visualize rabbit primary antibodies: Alexa Fluor donkey anti rabbit IgGs (H+L) 488, 594 or 647 (A21206, A21207, A31573 respectively, Molecular Probes) was used. To visualize mouse primary antibodies: Alexa Fluor F(ab')2 fragments of goat anti mouse IgG (H+L) 488, 594 or 647 (A11017, A11020, A21237 respectively, Molecular Probes) was used.

### Quantification of cell length

For quantification of the length and width of infected cells, nerves were first fixed and cryo-protected with successive glycerol baths. Nerves were then dissociated carefully to spread nerve fibres and allow clear visualization of the intact infected cells. These nerve spreads were cover-slipped in glycerol and imaged with confocal microscopy. This technique allowed optimal visualization of the intact infected cell while avoiding potential deformation that can result from teasing individual nerve fibres. The length and the diameter (mean value of 5–10 measures along the cell) of infected myelinating Schwann cells were measured according to the DsRed2 or GFP expression using ImageJ software. Demyelinating, non-myelinating or incomplete cells were not measured.

### Quantification of nuclear YAP immunostaining

Teased fibres immunostained for YAP were co-stained with nuclear DAPI or TOPRO3 (Thermofisher, France) dye to clearly define nuclei and to allow quantification of nuclear YAP immunostaining. Confocal Z-series of nuclear YAP immunostaining, DAPI/TOPRO3, and DsRed2 or GFP when cells were infected, were obtained using a Leica SP5 and a 40 × objective, ensuring no saturation of signal to allow subsequent accurate quantification. Stacked images (.tifs) were opened in ImageJ, the colour channels split and the scale set according to the embedded scale bar. The image containing DAPI was threshold corrected to clearly identify the full nucleus and a mask of all nuclei was generated. Using the ImageJ image calculator, the YAP staining channel ‘AND' the DAPI-nuclei mask were superposed, generating an image displaying only the YAP staining that was within the nuclei areas. All the nuclear-restricted YAP immunostaining was outlined (ROI) and the raw integrated densities and areas measured for each nucleus. The raw integrated densities were then normalized to the ROI areas to obtain an integrated YAP nuclear fluorescence density per μm^2^ for each nucleus. Using the channel containing DsRed2 or GFP, indicating infected cells, we then attributed the quantified YAP nuclear intensity to infected or non-infected cells on the same image. The presence of infected cells and non-infected cells nuclei on the same image ensured minimal variability in the quantification following immunochemistry. Quantifications were done with at least three injected nerves and at least three independent immunohistochemistry staining experiments. All immunostaining was conducted with relevant controls (no primary antibody included, no secondary included, control samples).

### Rat Schwann cell culture

RSCs were generated and cultured as described previously^55^,^56^. Briefly, passage 9 RSCs were grown on PDL to 80% confluence when they were infected with control GFP or YAP S127A Flag adenovirus (2 μl per 962 mm^2^) for 16 h in growth medium DMEM/F12 (D6421, Sigma) supplemented with 2 μm forskolin (F6886, Sigma) and 4 μg ml^−1^ bovine pituitary extract (BT215, Alfa Aesar). For differentiation, RSCs were growth-arrested in defined medium^56^ for 8 h and then supplemented with 1 mM dbcAMP (D0627, Sigma). Forty-eight hours later cells were scraped and protein extracted by sonication in standard RIPA buffer and processed for western blot.

### Western blot

Sciatic nerves were dissected from mice (C57 Bl6J, Janvier) at specified developmental ages. After removal of the epineurium and perineurium, the nerves were homogenized with a chilled mortar and pestle in standard RIPA lysis buffer. For protein extracts from whole sciatic nerves or RSCs, the protein concentration was measured using a BCA protein assay kit (Pierce). Between 10 and 25 μg proteins were directly analysed by western blot. Samples were processed using standard SDS–PAGE and western blotting procedures. Blots were digitized and analysed by densitometry with Quantity One software (BioRad).

Images in Figs 3a, 4d and 7a,c,e have been cropped for presentation and full size images are presented in Supplementary Figs 12–15, respectively. Images in Supplementary Figs 1, 3, 10, 11 have been cropped for presentation and full size images are presented in Supplementary Figs 16–19 respectively.

### Antibodies for western blots

Rabbit anti-ERLI (UM510) and Rabbit anti-CLPI (UM377, 1:250–500) were a kind gift from Dr Margolis, University of Michigan, USA).

Rabbit anti-pan-Crb3 (C2, kind gift from Dr A.Le Bivic, IBDML, France, 1:250)

Rabbit anti-YAP (#4912, Cell Signaling, 1:1,000)

Rabbit anti-phospho-YAP (S127A) (#4911, Cell Signaling, 1:1,000)

Rabbit anti-GFP (A11122, Invitrogen, 1:500)

Mouse anti-FLAG (F-3165, Sigma, 1:500)

Rabbit anti-MAG (#9086, Cell Signaling, 1:1,000)

Goat anti-MPZ (PA5-18773, Thermo Fisher, 1:3,000)

Rabbit anti-Rab11a (#2413, Cell Signaling, 1:1,000)

Rabbit anti-lamininγ1 (NBP1-19643, Novus Biologicals, 1:500)

Mouse anti-MBP (SMI94, Sternberger Monoclonal incorporated, 1:1,000)

Mouse anti-α-β-Actin (C1.AC-15, #A1978, Sigma Aldrich, 1:10,000)

Rabbit Pan-TEAD (D3F7L, Cell Signaling, 1:1,000)

Mouse anti-AKT (Cell signaling, 1:1,000)

Mouse anti-P-AKT (ser473, 587F11, Cell signaling, 1:1,000)

Secondary antibodies, Peroxidase Goat anti Rabbit or Mouse (H+L) (Jackson Immuno Research) were used at 1:10,000 dilution.

### Luciferase gene reporter assay

HEK 293T cells (27 × 10^3^) per well were seed in a 96-well plate in DMEM 10%FBS. Forty-eight hours later, cells were transfected in Optimem medium (Invitrogen) with 0.5 μl Lipofectamine 2000 (Invitrogen) and 0.6 μg DNA: 150 ng Renilla luciferase construct (pRL-CMV-Renilla, Promega), 150 ng Firefly luciferase constructs (pGL3 MPZ intron1 Addgene 21630 or pGL3Krox20 promoter Addgene 21260), 150 ng overexpressing construct 1 (pEGFP Clontech used as control, p2xFLAGhYAP1, Addgene 17791) and 150 ng of overexpressing construct 2 (pBluescript plasmid as carrier; pRK5-Myc-TEAD1 Addgene 33109; Myc-TEAD4 Addgene 24638). The Lipofectamine and DNA was incubated separately in Optimem medium (Invitrogen) for 5 min at 20–25 °C before mixing the two solution. The mix was incubated for 20 min at 20–25 °C before being added to the cells. After 4–6 h the mix is removed and fresh DMEM 10% FBS is added. Twenty-four hours after transfection, cells were lysed in 20 μl passive lysis buffer (Promega) and assayed for luciferase activity (Firefly and Renilla luciferase) using automated Varioskan Flash (Thermoscientific). Lysates were subjected to two consecutive injections of 75 μl of two different luciferase substrate and values were recorded after an integration time of 8 ms. Luciferase activity was normalized to Renillase activity. Results are expressed as a percentage of the GFP control value. Results shown are from three independent experiments in triplicates.

### Bone distraction

Adult SCID mice (Janvier, France) from 11 to 13 weeks old were first injected with 8 μl of adenovirus expressing or Crb3 sh3 or YAP sh1 in the sciatic nerve as described above. One week later these animals were anaesthetized again with isoflurane inhalation and the skin shaved and disinfected as described for the viral injection. A skin incision was made following the femur and muscles were also cut along this line to visualize the femur. A microTRACK alveolar distractor 9 mm (KSLmartin, 51-523-09-09) was modified by cutting the plate so only two screw holes remained on each side and slightly bent to conveniently adapt to the bone. The adjusted distraction apparatus was then fitted to the bone and fixed using two drill-free auto-blocking screws (diameter 0.9 mm; 5 mm long) placed on the last screw holes of the plate. The bone was then severed between the two screw fixations using a Gigli saw (0.22 mm, RISystem, Davos, Switzerland). The bone was then extended 0.6 mm apart using a special screwdriver (KLS Martin, 51-555-95-07). Each full turn of the screwdriver moved the distractor 0.3 mm. The muscles and the skin were then successively closed using absorbable sutures. Animals were treated with buprenorphine analgesic twice a day for 2 weeks. Every day, at regular hours, animals were anaesthetized using isoflurane and the distractor extended a further 0.6 mm. According to published works^30^,^57^ the rate of elongation we used did not induce any damage to the nerve and we did not detect any axonal thinning or axonopathy using immunostaining. At the end of the experiment animals were killed by CO_2_ inhalation and both elongated and non-elongated contralateral nerves were dissected and fixed in Zamboni's fixative for 10 min.

This protocol was adapted from previous publications^29^,^30^,^57^. We are grateful for RISystem (Davos, Switzerland) for their very useful online videos: (http://www.risystem.com/Standardized_Implant_for_Research/Training.html).

### Statistics

All results are presented as mean±s.e.m or s.d. All statistical analysis indicates *t*-test two-tailed *P* values. Significance: *P<0.05, **P<0.01, ***P<0.001; NS: not significant *P*>0.05.

### Data availability

The authors declare that the data supporting the findings of this study are available within the article and its Supplementary Information file.

## Additional information

**How to cite this article:** Fernando, R.N. *et al*. Optimal myelin elongation relies on YAP activation by axonal growth and inhibition by Crb3/Hippo pathway. *Nat. Commun.* 7:12186 doi: 10.1038/ncomms12186 (2016).

## Supplementary Material

Supplementary Information Supplementary Figures 1-19
