# Supplementary material for: Optimal myelin elongation relies on YAP activation by axonal growth and inhibition by Crb3/Hippo pathway
Source: Nat Commun. 2016 Jul 20;7:12186. doi: 10.1038/ncomms12186 (PMC4961766; doi:10.1038/ncomms12186)

Supplementary Fig. 1_Fernando et al

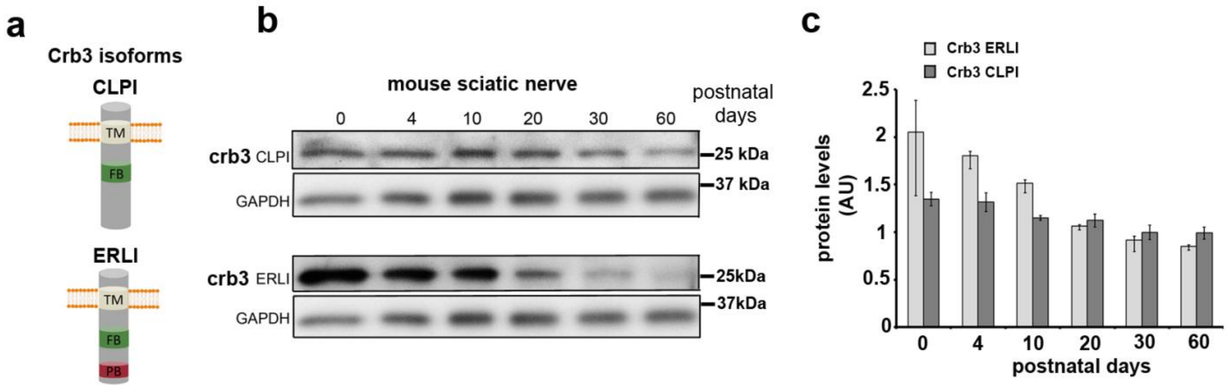

<u>**Supplementary Figure 1**</u> **Expression of Crb3 in mouse sciatic nerve: biochemical analysis** (**a**) Schematic of Crb3 isoforms, ERLI and CLPI, indicating the location of the transmembrane (TM), FRM binding (FB) and PDZ binding (PB) domains; Middle: Western blot analysis of Crb3 isoforms expression in mouse sciatic nerve at postnatal days 0, 4, 10, 20, 30 and 60. GAPDH is included as loading control. **(b)** Quantification of the relative expression of ERLI and CLPI during development in mouse sciatic nerve normalized to GAPDH. Error bars show s.d (n = 3 independent experiments).

Supplementary Fig. 2_Fernando et al

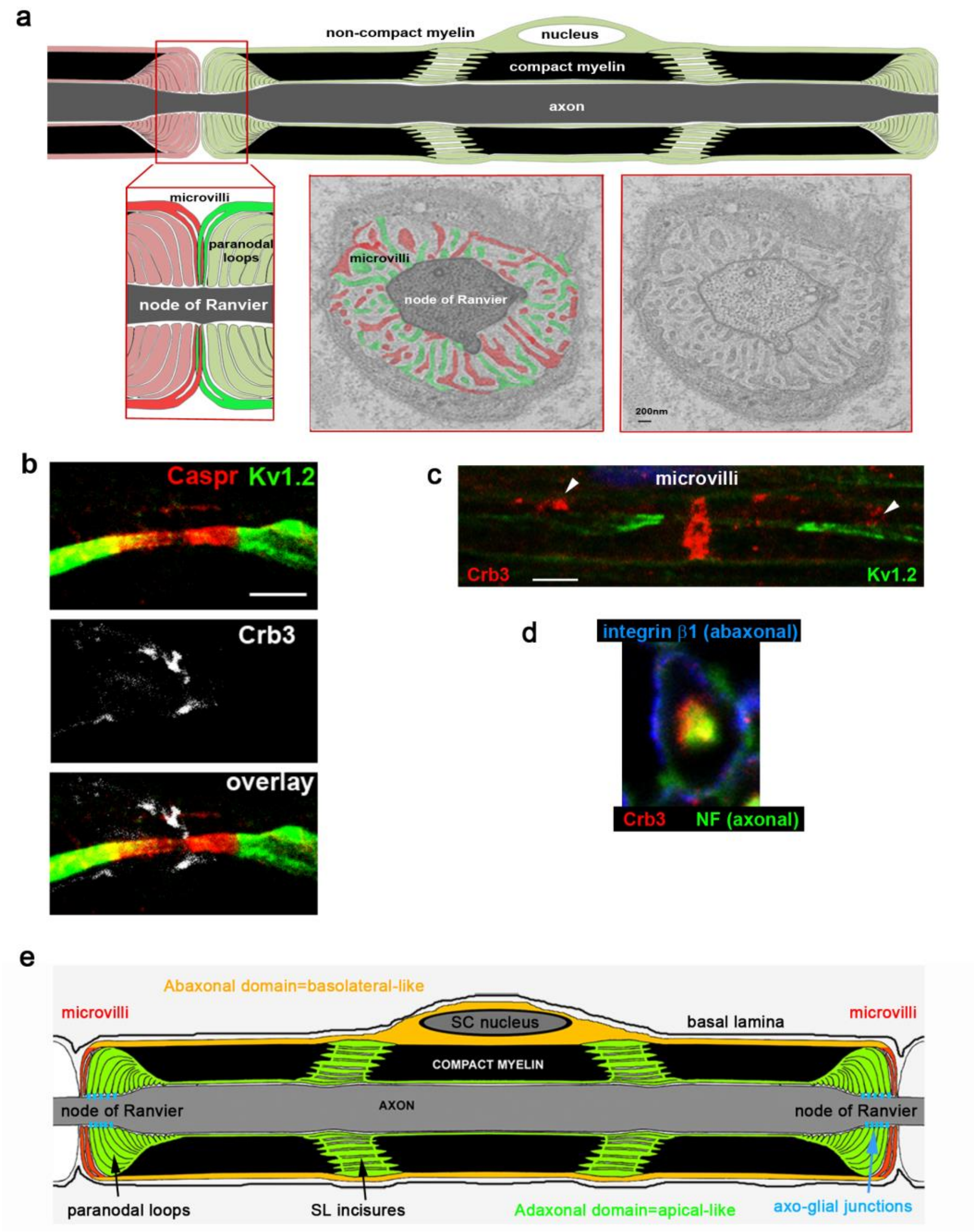

Supplementary Figure 2 Expression of Crb3 in mouse sciatic nerve (**a**) Schematic highlighting the location of Schwann cell microvilli. The microvilli are microscopic cellular membrane protrusions containing cytoplasm at the extremities of mSCs. They intermix with microvilli of adjacent cells (illustrated in red and green) and contact the node of Ranvier. Arbitrary pseudo-coloring has been used to highlight the microvilli from the adjacent cells in a cross section of a representative node of Ranvier electron microscopy image. (**b**) Single confocal plane of a node of Ranvier labelled with paranodal Capsr (red), juxtaparanodal Kv.1.1 (green) and Crb3 (white) showing that Crb3 staining is not on the axolemma. Scale bar: 10 µm. (**c**) Confocal image showing Crb3 staining (red) in the microvilli between two mSC and some minor staining in the adaxonal domain (arrowheads) close to the axon labelled with juxtaparanodal Kv1.2 staining (green). **(d)**. Mouse sciatic nerve cross-section labelled with Crb3 (red), axonal Neurofilaments (NF, Green) and Integrin β1 (blue) in the abaxonal domain of the mSC. Crb3 staining is localized in the adaxonal domain. Scale bars: 1µm. **(e)** Schematic drawing showing the structure of mSC around an axon, basal lamina, axo-glial junctions and the different polarity domains.

Supplementary Fig. 3_Fernando et al

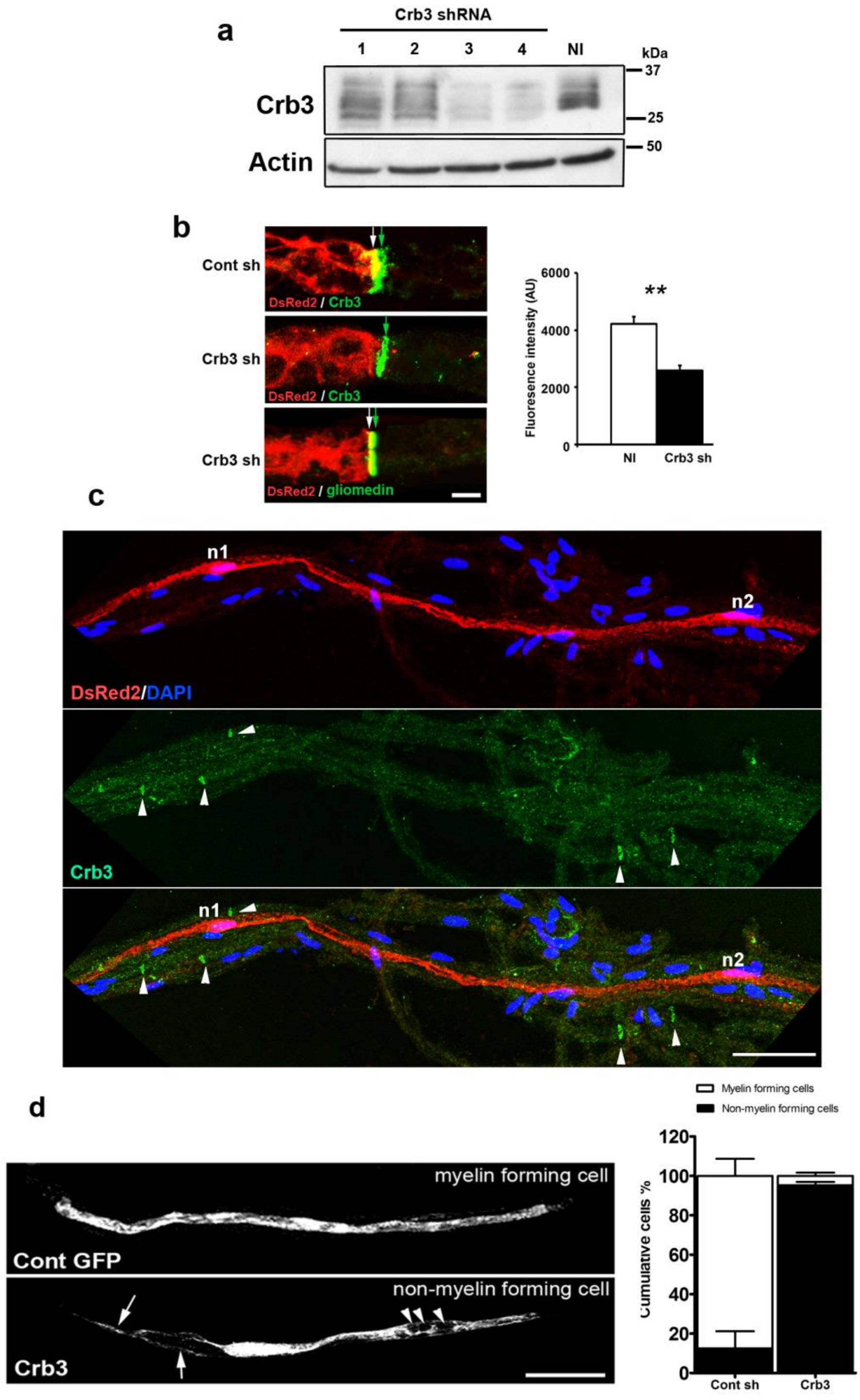

<u>**Supplementary Figure 3**</u> **Efficient silencing of Crb3 in cell lines and in mSCs in vivo**

(**a**) Western blot showing the reduction of Crb3 protein levels in EpH4-J3B1A cells infected with Crb3 shRNAs lentiviruses (3 and 4 both were used in all *in vivo* experiments). Multiple bands are due to posttranslational modifications of Crb3. NI: non-infected cells. **(b)** Crb3 expression is reduced in mSCs infected with Crb3 shRNA virus. **Left**: At nodes of Ranvier, Crb3 immunostaining represents shared microvilli of adjacent mSCs. **Top panel**: In a cell infected with control shRNA virus (DsRed2, red), Crb3 immunostaining (green) partially overlaps with cytoplasmic DsRed2 (yellow). The Crb3

immunostaining that overlaps with DsRed2 labelling localizes in the microvilli of the infected cell (white arrow) while the non-overlapping Crb3 immunostaining localizes in the microvilli of the non-infected cell (green arrow). **Middle panel**: In a cell infected with Crb3 shRNA virus (DsRed2, red), Crb3 immunostaining (green) does not overlap with cytoplasmic DsRed2, showing that Crb3 immunostaining is absent in the microvilli of the infected cell while it is still present in the microvilli of the non-infected cell (green arrow). **Lower panel**: In a cell infected with Crb3 shRNA virus (DsRed2, red), Gliomedin immunostaining (green) that labels microvilli, partially overlaps with cytoplasmic DsRed2 (yellow), showing that microvilli are still present in the infected cell (white arrow). Gliomedin immunostaining that does not overlap with DsRed2 localizes in the microvilli of the non-infected cell (green arrow). **Right**: Quantification of total Crb3 immunostaining between two non-infected cells (NI, n=13 nodes, 6 coverslips and 3 animals) or between a non-infected cell and a cell infected with Crb3 shRNA virus (Crb3 sh, n=16 nodes, 6 coverslips and 3 animals). On immunostained coverslips stacked Z-series confocal images (Leica SP5, 40X, without saturation) containing both infected and uninfected cells were obtained. In the same image, regions of interest (ROI) were selected encompassing the Crb3 immunostaining between two non-infected cells or between a non-infected cell and a cell infected with Crb3 shRNA virus (as seen in the left, middle panel). For each ROI the raw integrated density was measured using ImageJ software and normalized to the ROI area. Crb3 immunostaining is significantly decreased in microvilli adjacent to a Crb3 silenced cell (Statistical two-tailed Student's T-test: **$p<0.01$). Error bars show s.e.m. AU: Arbitrary unit. NI: Not infected. **(c)** Two successive mSCs (n1 and n2 showing their DAPI-labelled nuclei) infected with a virus expressing Crb3 shRNA1 and Dsred2 (red) show no Crb3 staining (green) between them. Clear Crb3 microvilli staining is seen between surroundings cells as a band pattern (arrowheads). (**d**) **Left**. a mSC infected with control adenovirus expressing GFP alone (Cont GFP) displays a myelinating phenotype with homogenous thickness and continuous cytoplasm (top panel). A cell infected with adenovirus expressing Crb3 and Dsred2 (Crb3, bottom panel) displays the hallmarks of a demyelinating phenotype, including string-like appearance of the cell at the places where the compact myelin is absent (arrows) and beaded structures in areas where compact myelin remains (arrowheads). Scale bars, 50 µm. **Right**. The percentage of non-myelin-forming cells among infected cells increases in nerves injected with a virus expressing Crb3 compared to nerves injected with a virus expressing GFP alone. All infected cells found in injected nerves were counted as myelinating or non-myelin forming (demyelinating Schwann cells, non-myelinating Schwann cells or fibroblasts) and their respective numbers expressed as a percentage of the total cell number. Number of nerves analyzed: 4 (GFP), 6 (Crb3). Error bars show SEM. Scale bars, 200 nm (**b**), 50 µm (**c**).

Supplementary Fig. 4_Fernando et al

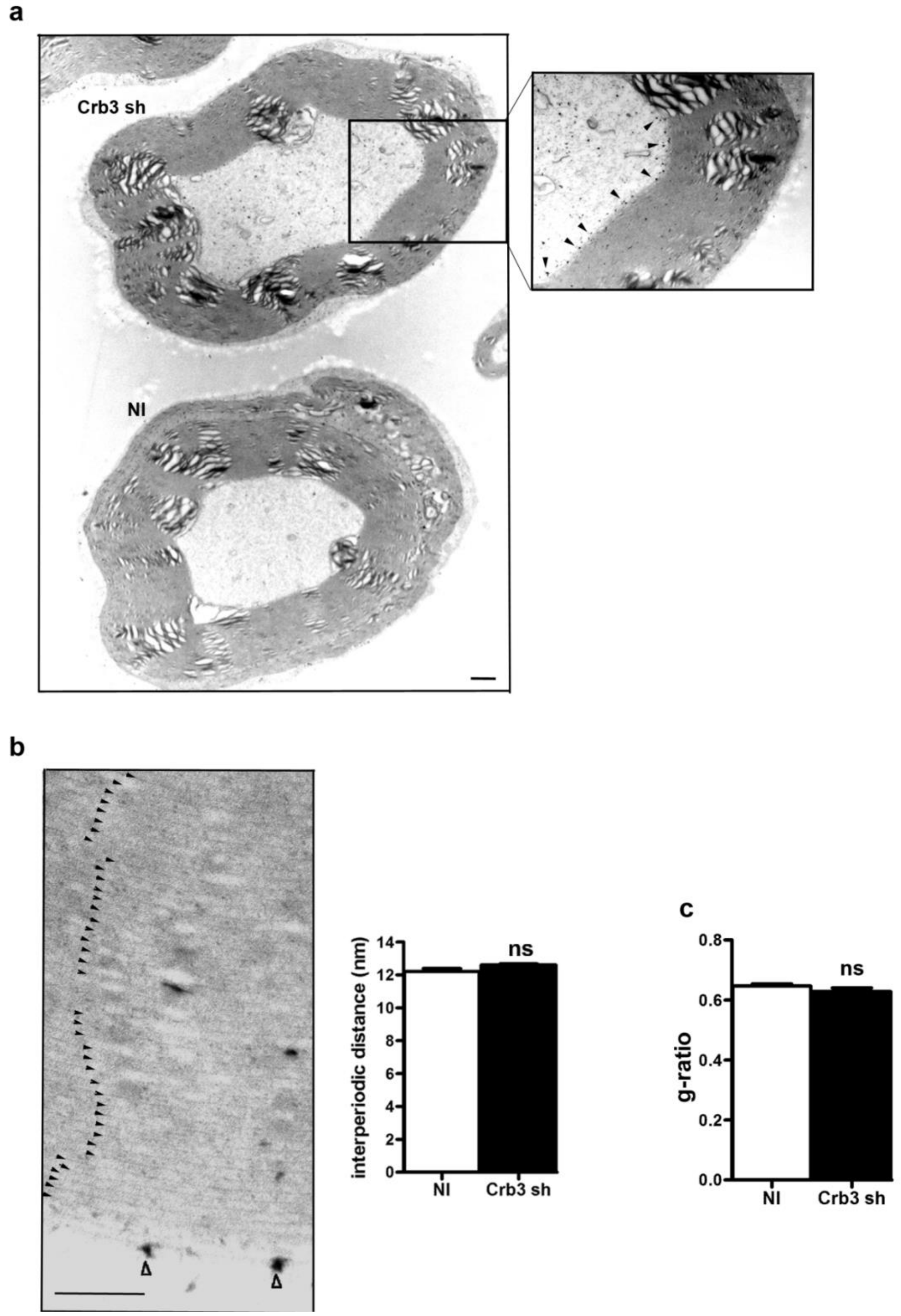

<u>Supplementary Figure 4</u> **Crb3-silencing does not alter the myelin ultrastructure, interperiodic distance or the myelin g-ratio (a)** Electron microscopy picture showing two adjacent myelinated fibers in a cross-section from a 2 month old mouse sciatic nerve infected with Crb3 shRNA PLAP virus. The upper mSC displays small black precipitates at the interface with the axon indicating this cell expresses PLAP enzyme and is infected with the virus (Crb3 sh). The adjacent mSC does not show black precipitate and is not infected (NI). The insert on the right shows an enlargement of the infected cell highlighting the precipitates (arrowheads). (**b**) Left: Electron microscopy picture showing a detail of the compact myelin of a mSC infected with Crb3 shRNA PLAP virus in a cross-section from a 2 month old mouse sciatic nerve. The interperiodic lines are indicated with black arrowheads. Note the black precipitates resulting from PLAP activity at the interface with the axon in the lower part of the picture (clear arrowheads). Right: The distance between two interperiodic lines (interperiodic distance) was measured for 11 Crb3-silenced cells (242 interperiodic lines, Crb3 sh) and 5 non-infected cells (82 interperiodic lines, NI). No significant change is observed (two-tailed Student's T-test: P>0.05; 4 animals). (**c**) The g-ratio was calculated for 90 infected mSCs (Crb3 sh) and 341 non-infected mSCs (NI) by measuring axonal and total fiber perimeters. No significant change is observed (Statistical Student's T-test: ns, P>0.05; 4 animals).Scale bar, 500 nm (**a**) 100 nm. (**b**) Error bars show s.e.m.

Supplementary Fig. 5_Fernando et al

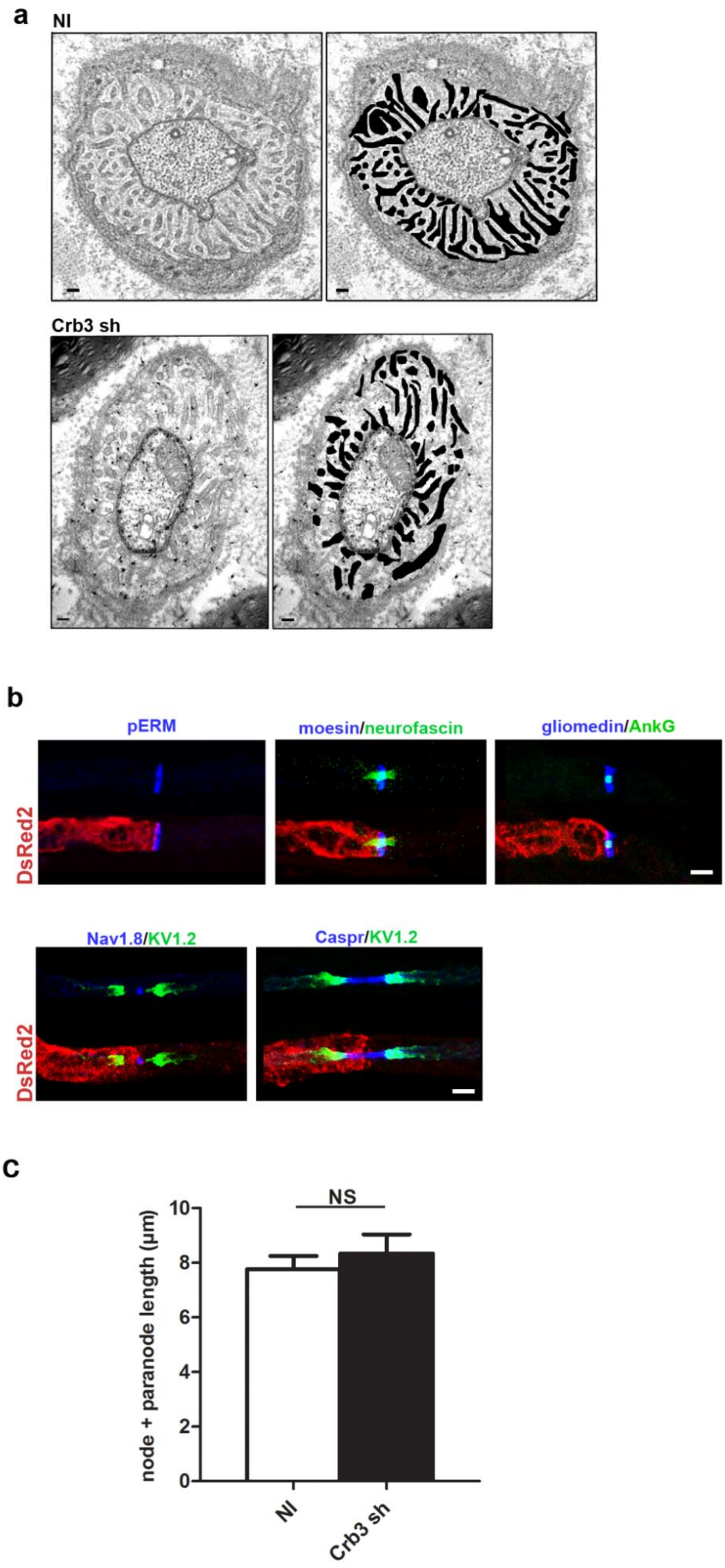

<u>Supplementary Figure 5</u> Microvilli and nodes of Ranvier are not altered in Crb3-silenced mSCs (**a**) Left panels: electron microscopy pictures showing microvilli (highlighted in black in the right panels) of a non-infected mSC (NI) or a mSC infected with Crb3 shRNA PLAP virus (Crb3 sh) around a node of Ranvier. In both pictures microvilli show a similar diameter and make contact with the axonal membrane. (**b**) All panels focus on nodes of Ranvier between a mSC infected with Crb3 shRNA virus (DsRed2, red) and a non-infected adjacent cell. These nodes are labelled with microvilli markers, pERM, Moesin and Gliomedin (blue), and with nodal markers Neurofascin (green), Ankyrin G (AnkG, green) and Nav1.8 (blue), and with juxtaparanodal KV1.2 (green) and paranodal Caspr (blue) markers. The same image, without DsRed2, is offset above to allow clear visualization of the markers in infected and non-infected cells. All markers are present and localized as reported in the literature. Their distributions appear symmetrical between the Crb3-silenced cell and the non-infected cell, suggesting that Crb3 silencing does not affect microvilli and nodes of Ranvier marker expression or distribution. (**c**) Quantification of node+paranode length using Pan-neurofascin, Caspr and KV1.2 immunostaining in nodes between one Crb3-silenced cell and a non-infected cell (Crb3 sh) or in nodes between two non-infected cells (NI). Error bars show s.e.m. No significant change is observed (Statistical Student's T-test: ns, $p > 0.1$; $n=17$ (Crb3 sh), 30 (NI); 3 animals). Scale bars, 100 nm (**a**), 5 µm (**b**).

Supplementary Fig. 6_Fernando et al

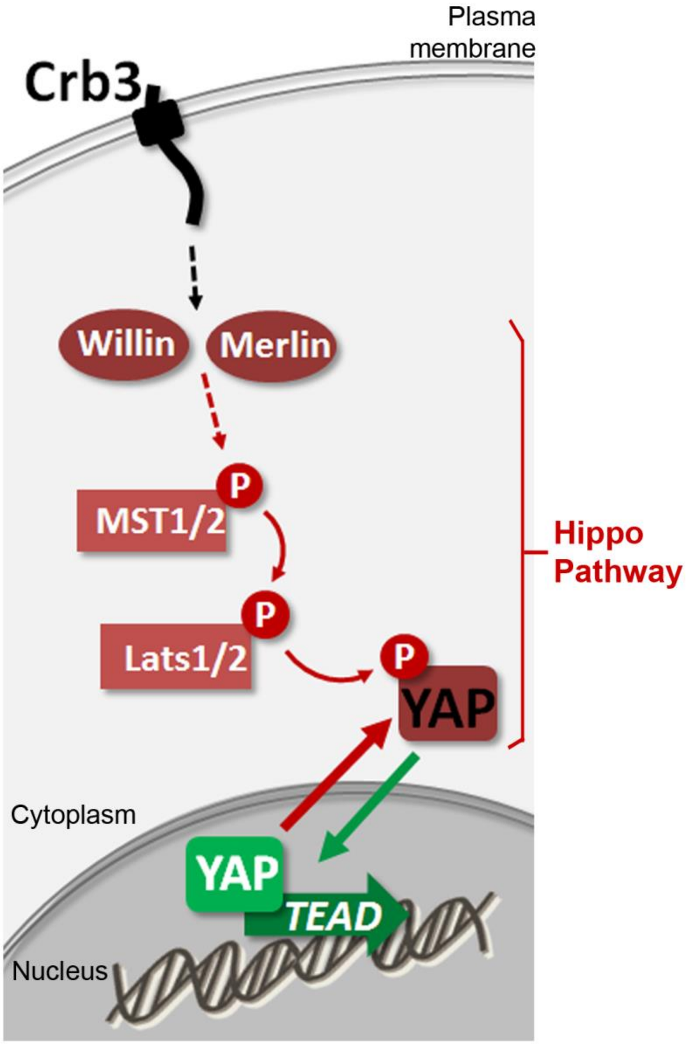

<u>Supplementary Figure 6</u> Schematic of major components of the mammalian Hippo pathway. In mammalian cells the Hippo pathway negatively regulates cell growth by preventing the nuclear localization of the transcriptional cofactor YAP. Crb3 is proposed to be the plasma membrane receptor, which signals through Willin and/or Merlin (black dotted arrow) to activate a conserved phosphorylation cascade of MST1/2 and Lats1/2 (red arrows; phosphorylation is illustrated with a circled P which in turn can phosphorylate YAP leading to its cytoplasmic retention (thick red arrow). When the Hippo pathway is not active YAP can enter the nucleus (green arrow) where it interacts with TEAD transcription factors promoting the transcription of genes (large green arrow) involved in cell and tissue growth and in anti-apoptotic mechanisms.

Supplementary Fig. 7_Fernando et al

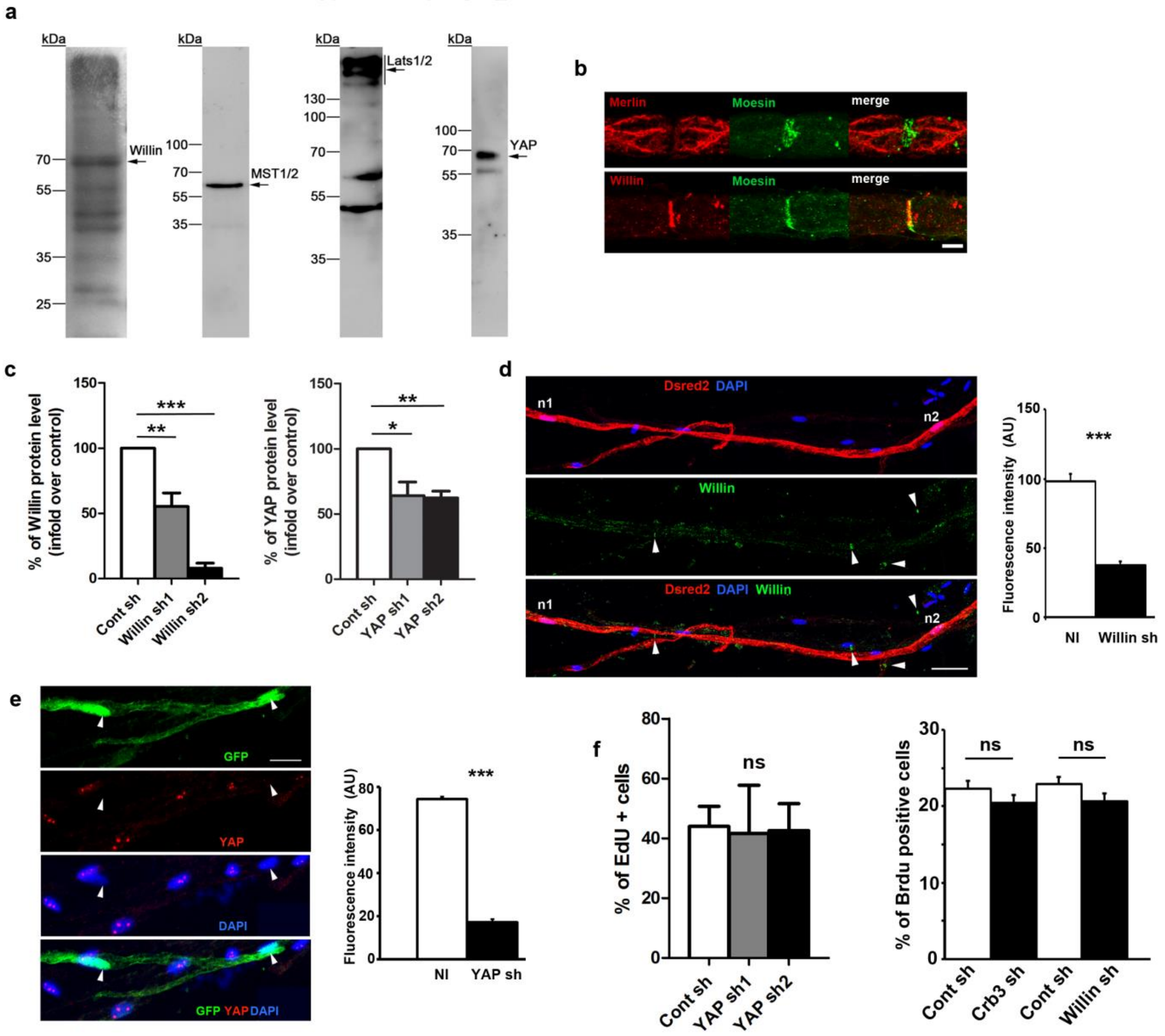

Supplementary Figure 7 **Expression of major components of the Hippo pathway in peripheral nerves**. **(a)** Western blots of sciatic nerves lysates from 30 day old mouse were separated by SDS-PAGE. Antibodies specific to Willin (~72 kDa), MST1/2 (~60 kDa), Lats1/2 (~ 140 kDa) and the transcriptional target of the pathway, YAP (~ 65 kDa), were hybridized overnight and bound antibodies were detected with secondary antibodies coupled to HRP. Last1/2 and Willin antibodies show non-specific bands at lower weights. These data confirms the expression of key Hippo pathway components in the sciatic nerve of mice. **(b)** Merlin (red, top panel) is expressed in the Cajal's bands of mSC and does not colocalize with microvilli labelled with Moesin (green). In contrast, Willin (red, bottom panel) is expressed in microvilli and colocalize with Moesin (green). (**c**) Quantification of Willin (left) and YAP (right) expressions in MSC80 cells transfected with plasmids expressing or Control shRNA (Cont sh) or Willin shRNA1 and 2 (Willin sh1 and sh2) or YAP shRNA 1 and 2 (YAP sh1 and sh2) respectively. Western blots were used to detect Willin and YAP in solubilized MSC80 cells and Actin was used as loading control for normalization. Willin shRNA 1 and 2 and YAP shRNA 1 and 2 significantly reduce Willin and YAP expression respectively (P = 0.006, 0.0008, 0.01, 0.009

respectively; n =3 independent experiments). (**d**) **Left**. Two successive mSCs (n1 and n2 showing their DAPI-labelled nuclei) infected with a virus expressing Willin shRNA2 and Dsred2 (red) show no Willin staining (green) between them. Clear Willin microvilli staining is seen between surroundings cells as a band pattern (arrowheads). **Right.** Quantification of total Willin immunostaining between two non-infected cells (NI) or between a non-infected cell and a cell infected with Willin shRNA2 virus (Willin sh) as described in legend Supplementary Fig. 2b. Willin immunostaining is significantly decreased in microvilli adjacent to a Willin silenced cell ($P < 0.001$). Error bars show s.e.m. Results comprise 33 (NI) and 30 (Willin sh) nodes from 8 coverslips (3 animals). (**e**) **Left**. Two mSCs with their DAPI-labelled nuclei (Arrowheads, blue) are infected with a virus expressing YAP shRNA1 and GFP (green) and show no nuclear YAP staining (red). Surrounding non infected cells have YAP staining in their nuclei. **Right.** Quantification of total YAP immunostaining in the nuclei of non-infected cells (NI) or of cells infected with YAP shRNA1 virus (YAP sh) as described in the methods. YAP immunostaining is significantly decreased in the nuclei of YAP silenced cell ($P < 0.001$). Error bars show s.e.m. Results comprise 29 (NI) and 47 (YAPsh) nuclei from 8 coverslips (3 animals). (**f**) **Left.** MSC80 cells were transfected with Control ShRNA + GFP (Cont sh), YAP shRNA1 + Dsred2 (YAP sh1) or YAP shRNA2 + Dsred2 (YAP sh2) expressing plasmids using Lipofectamine 2000 (Invitrogen). After 24 hrs cells were split and seeded in triplicate on glass coverslip. 24 hours later cultures were incubated with 10µm EdU (Baseclick, Germany) for 6 hours, washed in PBS and fixed in 4% paraformaldehyde. We then used the Click-it kit (Baseclick, Germany) or with green Alexafluor 488 or red 594 to detect EdU. The percentage of EdU positive cells among the transfected cells (detected with the fluorescent probes) is plotted in the graph. No significant change is observed between Cont sh and Crb3 sh1 and Cont sh and Crb3 sh2 ($P = 0.82$ and 0.83; n=3 independent experiments and at least 342 cells counted for each condition). **Right.** Mouse pups were injected at P3 with virus expressing control shRNA (Cont sh), Crb3 shRNA (Crb3 sh) or Willin shRNA (Willin sh) and injected at P5 intraperitoneally with 100mg/kg of BrdU. One week later animals were sacrificed and longitudinal sections of the injected sciatic nerves were processed as described previously[56]. The percentage of BrdU positive nuclei among DAPI labelled nuclei is plotted in the graph. No significant change was observed (n=3 mice and between 111 and 251 nuclei counted per mice). Error bars show s.e.m.

Otherwise indicated (d) all error bars show s.d. All statistical P-values are Student's T-test. ns: non-significant. Scale bars= 5 µm (b), 100 µm (d and e). AU: Arbitrary unit.

Supplementary Fig. 8_Fernando et al

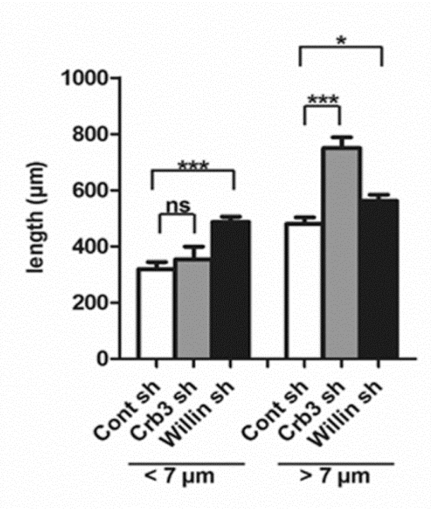

<u>Supplementary Figure 8</u> Crb3 silencing affects large caliber fibers while willin silencing affects more significantly small caliber fibers

Data collected for Fig.1f and 2b were plotted relatively to fiber diameter >7µm or < 7µm. Error bars show s.e.m. Statistical two-tailed Student's T-test: * P<0.05; ** P<0.01; *** P<0.001. ns: non-significant P>0.05.

Supplementary Fig. 9_Fernando et al

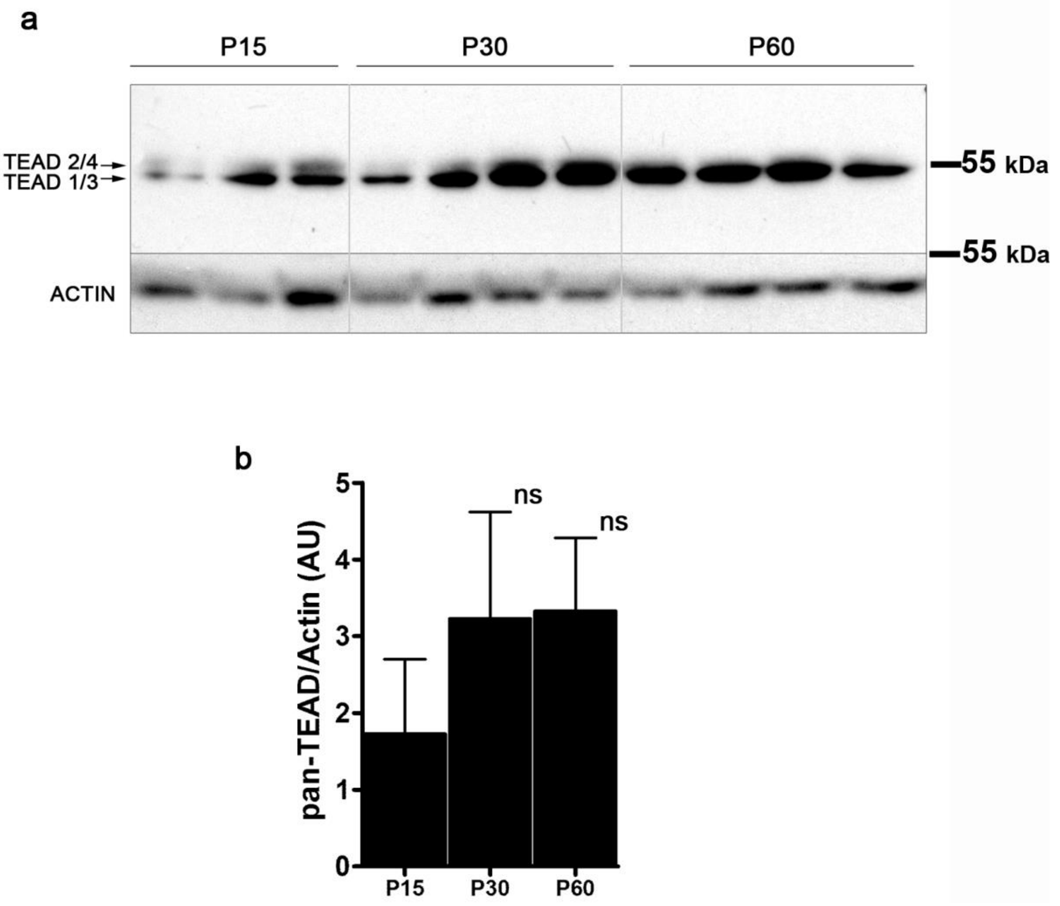

<u>Supplementary Figure 9</u> **Expression of TEAD factors in Sciatic nerve.** (**a**) Western blot analysis of TEAD factors expression in mouse sciatic nerve at 15, 30 and 60 dPN (P15, P30, P60). We used a pan-TEAD antibody so all TEADs are detected. Accordingly to the literature and the antibody provider the upper band contains TEAD 2 and 4 and the lower TEAD 1 and 3. Actin is included as loading control. (**b**) Quantification of the relative expression of all TEAD factors at each time point normalized to Actin. Error bars show s.d (n = 3 independent experiments; statistical two-tailed Student's T-tests P-value>0.05, ns: non-significant).

Supplementary Fig. 10_Fernando et al

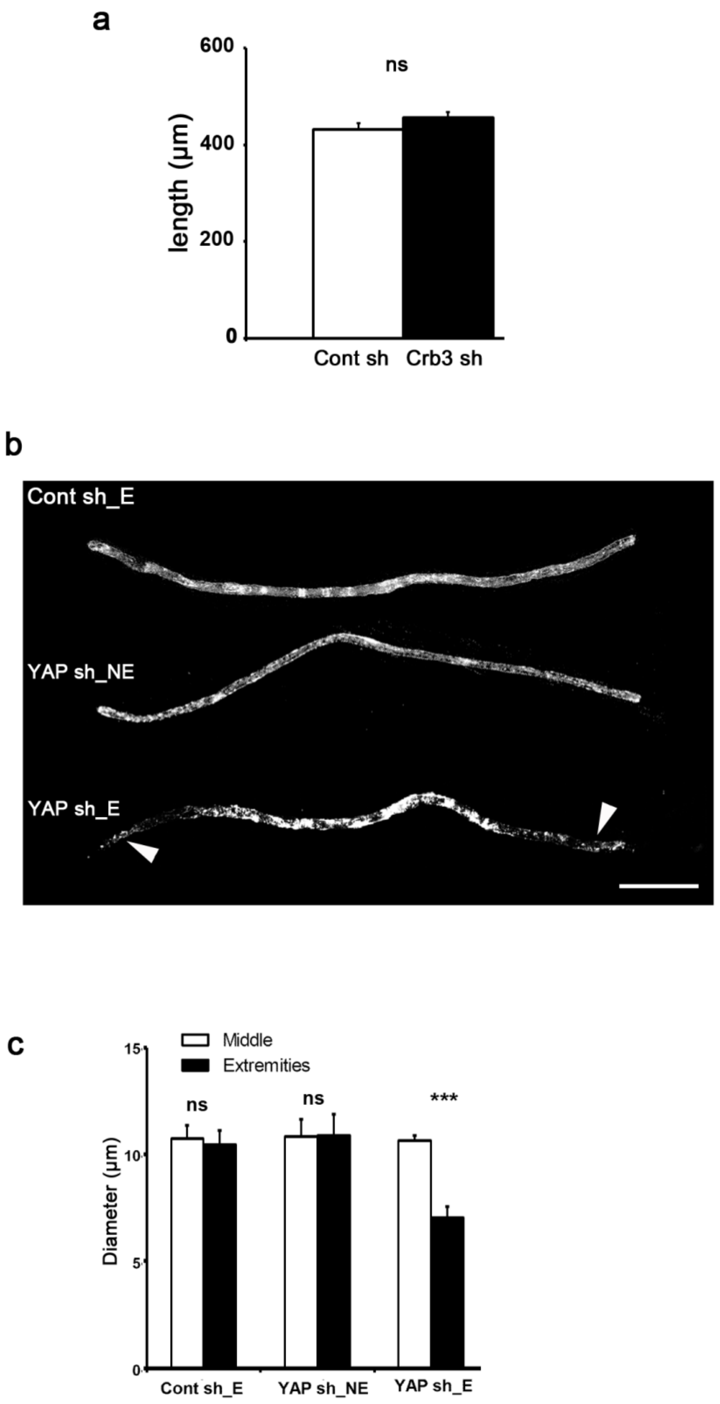

Supplementary Figure 10

Crb3 silencing in adult mice does not increase myelin length and YAP-silenced mSCs do not elongate in artificially extended nerve (**a**) In adult mice (11-13 weeks old) mSCs infected with a virus expressing Crb3 shRNA1 (Crb3 sh) are not longer than cells infected with a control shRNA virus (Cont sh). ns: non-significant. n= 106 (Cont sh; 3 animals), 123 (Crb3 sh; 3 animals) cells. (**b**) A representative cell from an artificially elongated nerve infected with YAP shRNA virus (YAP sh_E, GFP, bottom) is compared to a cell infected with YAP shRNA from a non-elongated nerve (YAP sh_NE, GFP, middle) and to a cell infected with Cont sh virus in an elongated nerve (Cont sh_E, GFP, top). Note the heterogeneous structure of the YAP sh E cell (arrows). Scale bar, 100 µm. (**c**) Quantification of the average diameter of each cell type described in (**b**) at the middle and at the extremities (20 µm from the cell ends). Mean of at least 5 measures at the extremities and at the middle. Student T-test statistical analysis compares extremity diameters to middle cell diameters for Cont sh E, YAP sh NE and YAP sh E. n = 31 (3), 35 (3), 35 (3) cells (animals) respectively. Extremities diameter is significantly smaller than middle diameter in YAP sh E cells but not in Cont sh E or in YAP sh NE cells. Error bars show s.e.m.

Supplementary Fig. 11_Fernando et al

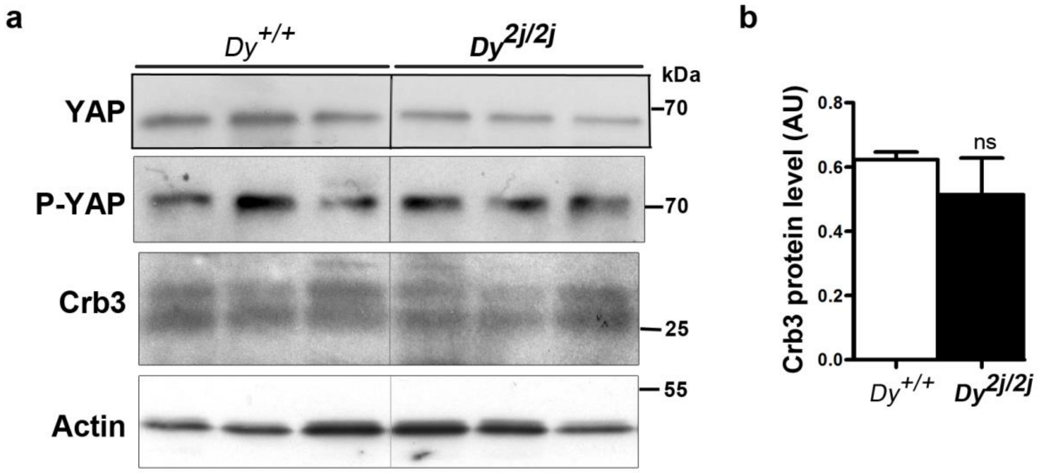

<u>**Supplementary Figure 11**</u> **YAP, phosphorylated YAP and Crb3 expression is sciatic nerves of *Dy*$^{+/+}$ and *Dy*$^{2j/2j}$ mice.** (**a**) Western blots of 15dPN sciatic nerve preparations from *Dy*$^{+/+}$ and *Dy*$^{2j/2j}$ (3 individual littermates) for Crb3 (~ 25-35 kDa), YAP (~ 70 kDa) and phosphorylated YAP (P-YAP; ~ 70 kDa). Actin (~ 48 kDa) was used to verify loading. Quantification of total YAP and P-YAP protein levels, normalized to actin, are in **Fig. 8b** and **c** respectively. (**b**) Quantification of total Crb3 protein levels from the Western blots shown in (**a**), normalized to actin. Error bars show s.d. Statistical two-tailed Student's T-test P = 0.212. ns: non-significant.

Supplementary Figure 12: Full blots Fig. 3a

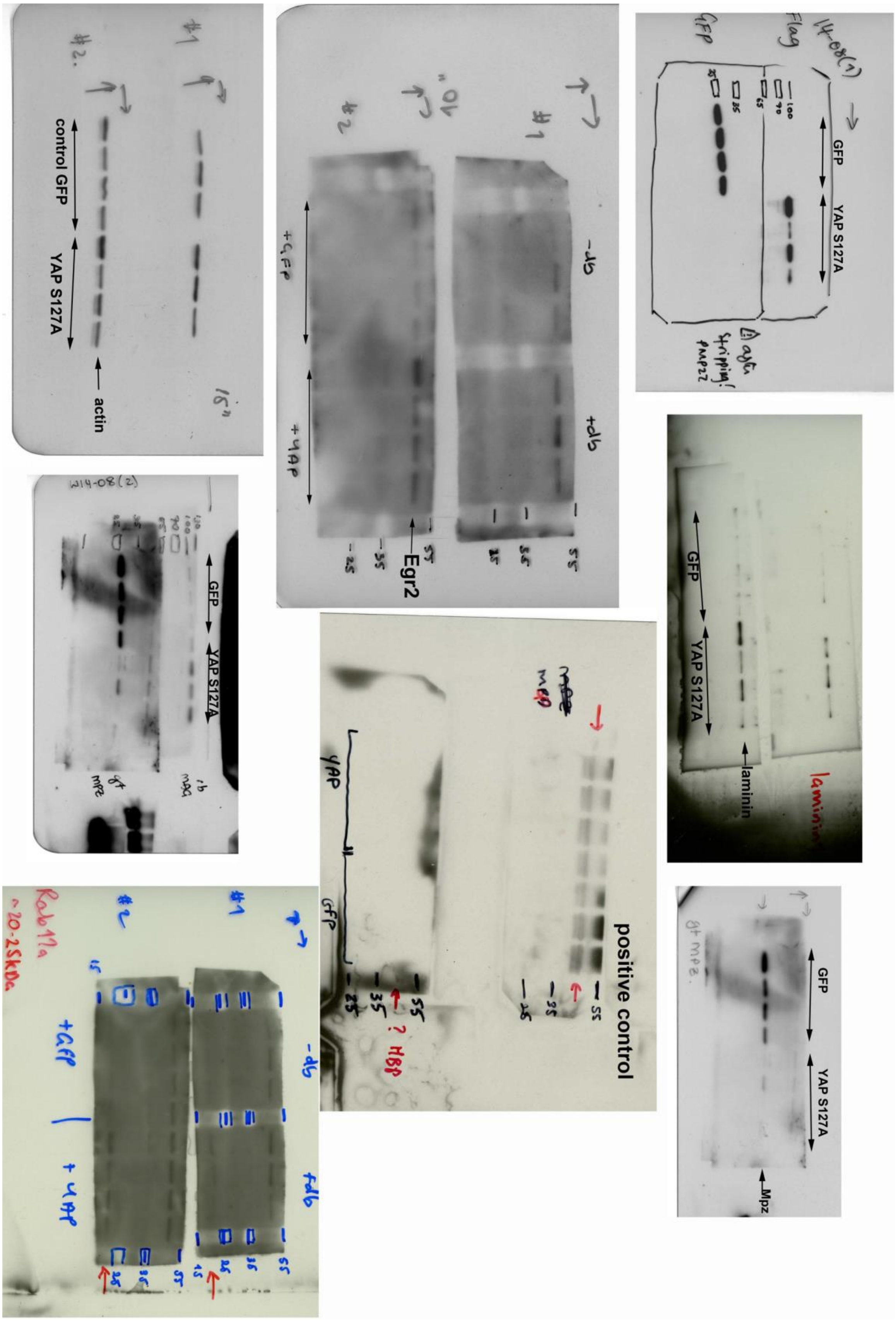

Supplementary Fig. 13:

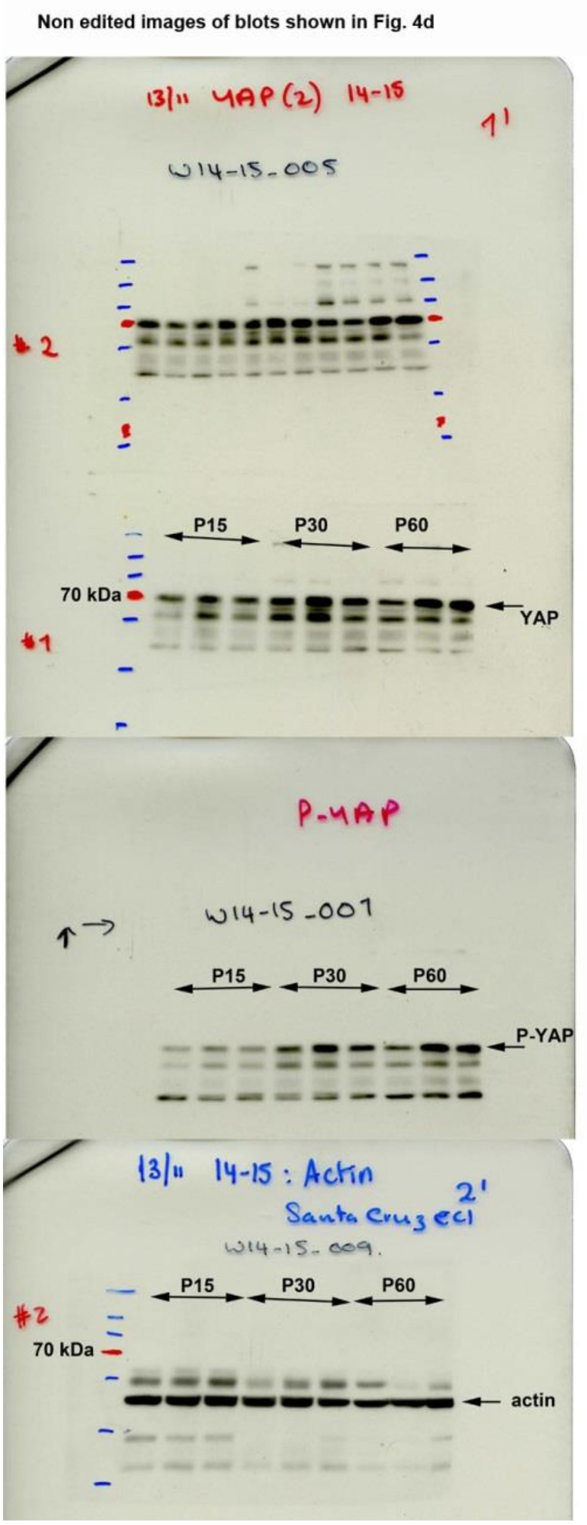

Supplementary Fig. 14: Full blots Fig. 7a

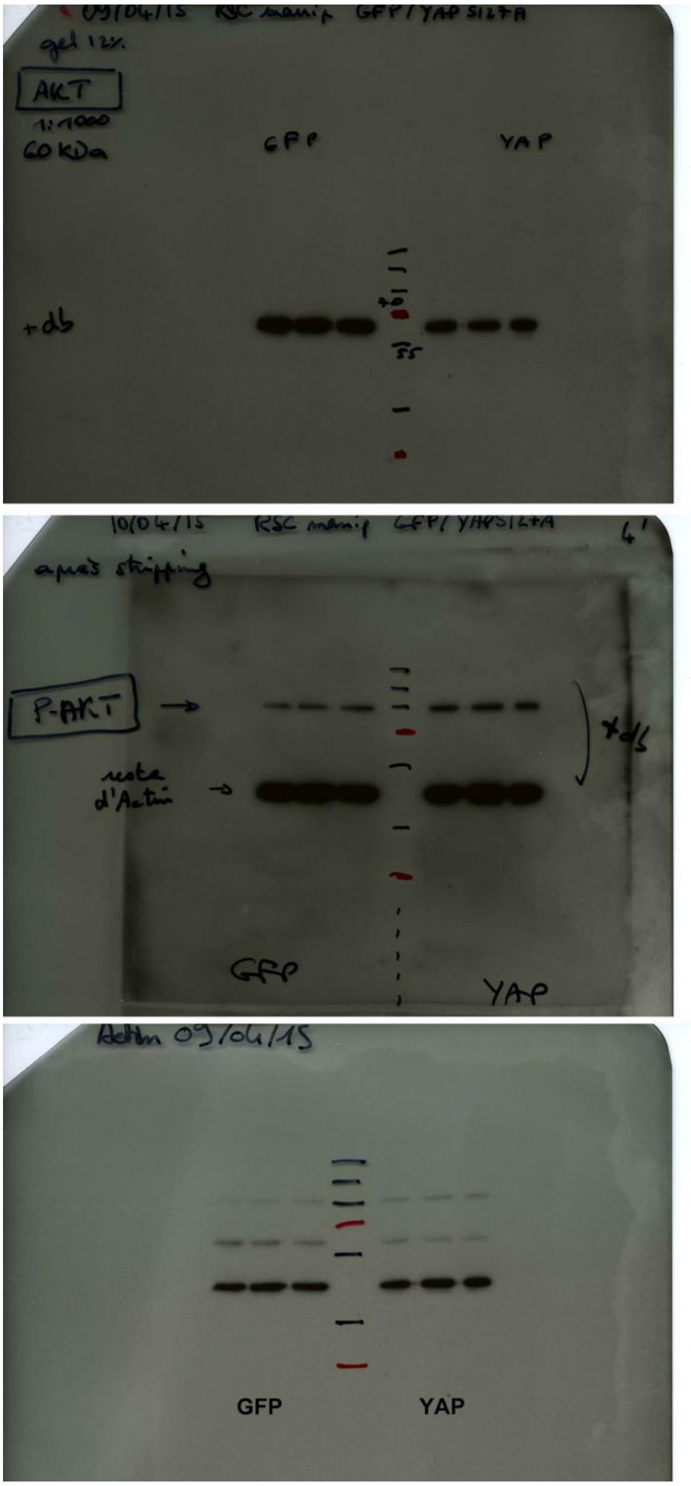

Supplementary Fig. 15: Full blots Fig.7c and e

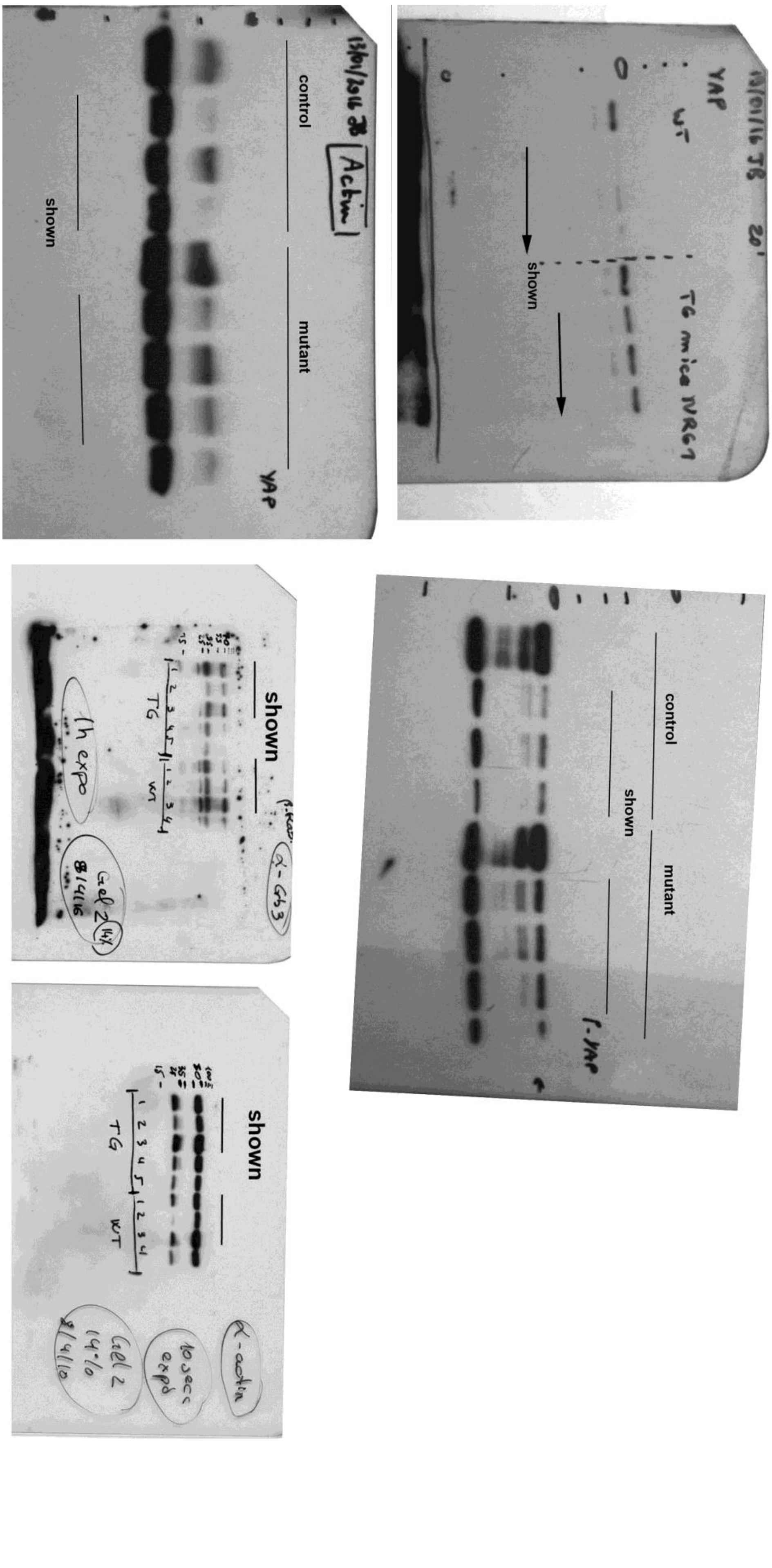

Supplementary Fig. 16: Full blots Supplementary Fig.1

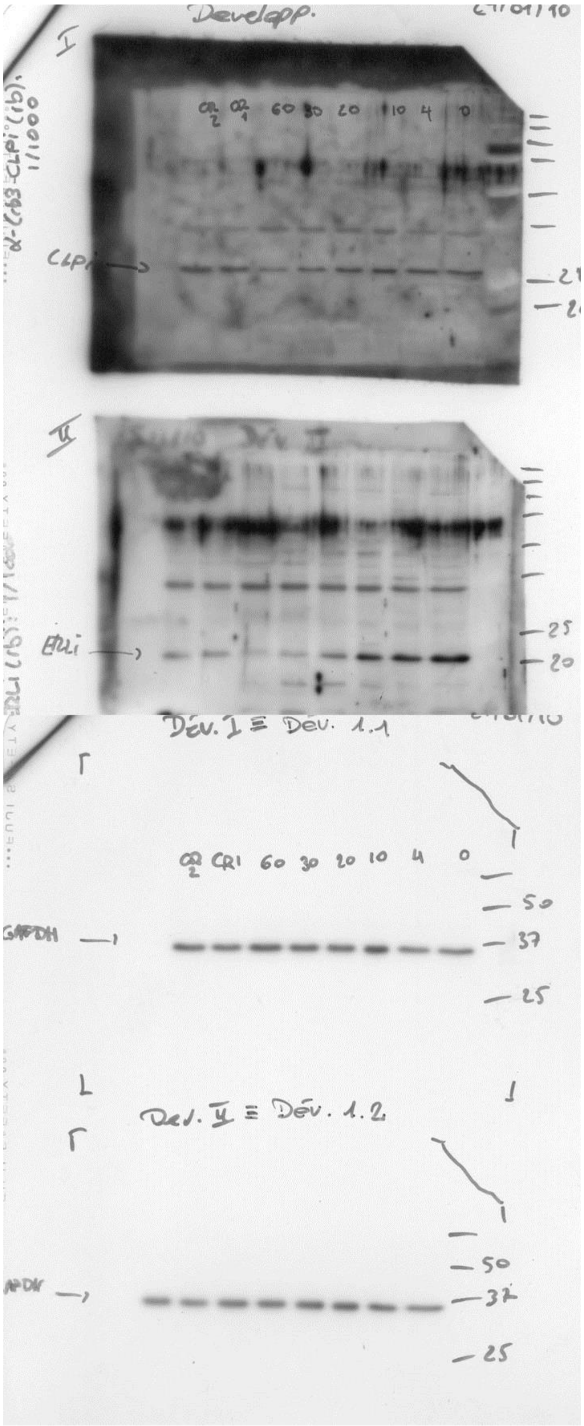

Supplementary 17: Full blots Supplementary Fig.3

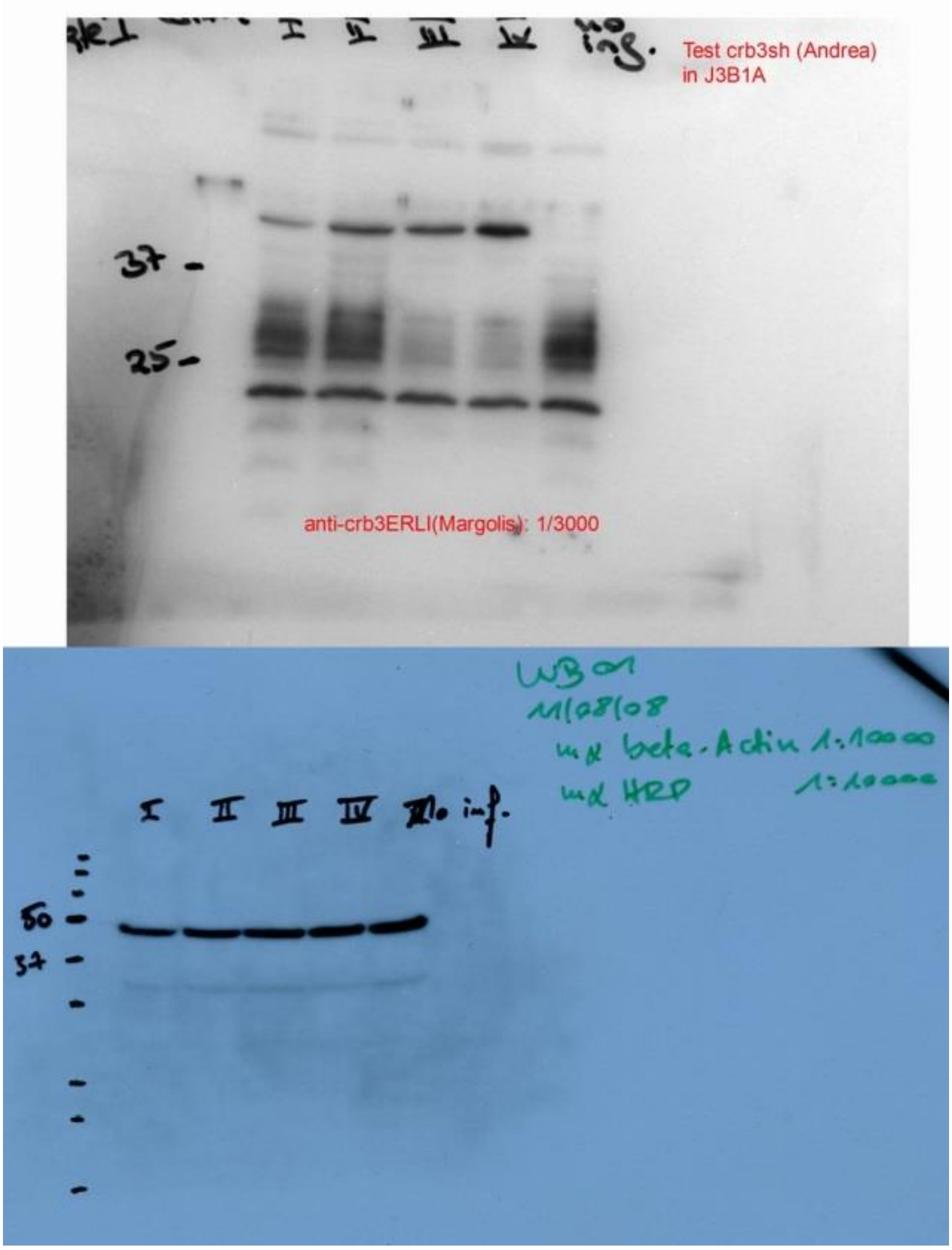

Supplementary Fig. 18: Full blots Supplementary Fig. 9

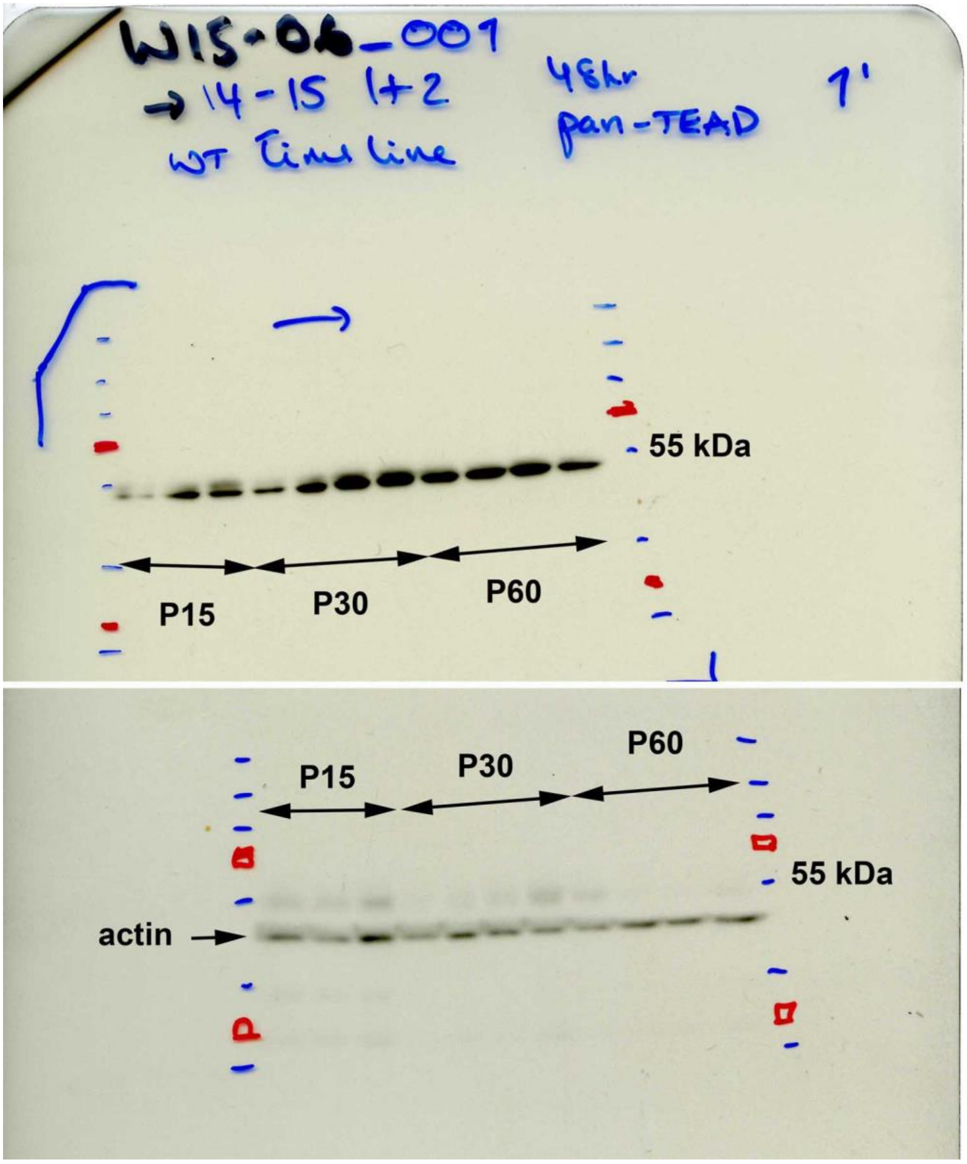

Supplementary Fig. 19: Full blots Supplementary Fig. 10

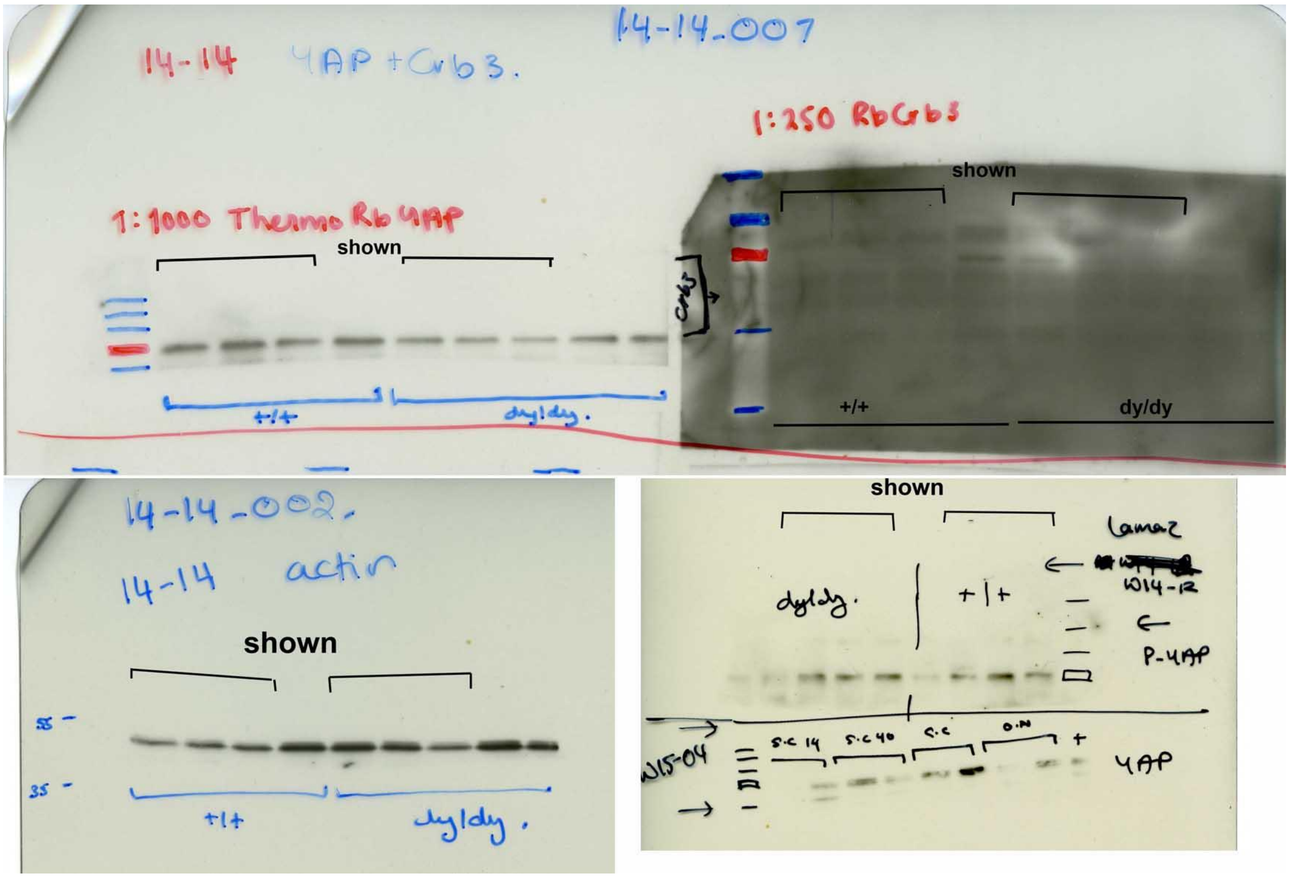

Supplement: Supplementary Information — Supplementary Figures 1-19 [file ncomms12186-s1.pdf]
